# High residual cardiovascular risk after lipid-lowering: prime time for Predictive, Preventive, Personalized, Participatory, and Psycho-cognitive medicine

**DOI:** 10.3389/fcvm.2023.1264319

**Published:** 2023-10-16

**Authors:** E. Reijnders, A. van der Laarse, J. W. Jukema, C. M. Cobbaert

**Affiliations:** ^1^ Department of Clinical Chemistry and Laboratory Medicine, Leiden University Medical Center, Leiden, Netherlands; ^2^ Department of Cardiology, Leiden University Medical Center, Leiden, Netherlands; ^3^ Netherlands Heart Institute, Utrecht, Netherlands

**Keywords:** residual cardiovascular risk, residual inflammatory risk, residual thrombotic risk, personalized medicine, p5 medicine, precision medicine

## Abstract

As time has come to translate trial results into individualized medical diagnosis and therapy, we analyzed how to minimize residual risk of cardiovascular disease (CVD) by reviewing papers on “residual cardiovascular disease risk”. During this review process we found 989 papers that started off with residual CVD risk after initiating statin therapy, continued with papers on residual CVD risk after initiating therapy to increase high-density lipoprotein-cholesterol (HDL-C), followed by papers on residual CVD risk after initiating therapy to decrease triglyceride (TG) levels. Later on, papers dealing with elevated levels of lipoprotein remnants and lipoprotein(a) [Lp(a)] reported new risk factors of residual CVD risk. And as new risk factors are being discovered and new therapies are being tested, residual CVD risk will be reduced further. As we move from CVD risk reduction to improvement of patient management, a paradigm shift from a reductionistic approach towards a holistic approach is required. To that purpose, a personalized treatment dependent on the individual’s CVD risk factors including lipid profile abnormalities should be configured, along the line of P5 medicine for each individual patient, i.e., with Predictive, Preventive, Personalized, Participatory, and Psycho-cognitive approaches.

## 1. Introduction

In the past decennia of medical literature, the term “residual cardiovascular risk” has hardly been defined and its meaning has changed repeatedly. The era of statin therapy has given a new meaning to the understanding of this term: residual CVD risk is often defined as the risk of CVD despite statin therapy according to current guidelines. This review about residual CVD risk describes the definitions used for this term, and the factors underlying this risk. As we mention the therapies to modify residual CVD risk by medical and lifestyle measures, the review ends with recommendations for personalized treatment of any individual or patient with a risk of residual CVD.

### 1.1. Definition

Most often the definition of residual CVD risk is coined to the risk of an individual having a major adverse coronary event (MACE) due to coronary artery disease (CAD) that has an atherosclerotic, inflammatory, or thrombotic cause despite therapy. As new CV risk factors are discovered, the definition of residual CVD risk has evolved over time. To our knowledge the first defined mention of residual CVD risk was in 1985 by Beaumont et al. who described residual vascular risk after discontinued oral contraception (1). Later on, it is described as the risk of CVD despite antihypertensive therapy. With the upcoming popularity of successful statin therapy to decrease low-density lipoprotein-cholesterol (LDL-C) levels, the definition of residual CVD risk shifted towards the risk of CVD despite statin therapy, and -in the mid 2000’s- the residual CVD risk after achieving target LDL-C levels during statin therapy. At that time, it was thought that a majority (≈70%) of the individuals who were treated with statins have a substantial risk of MACE (2). From 2010 onwards, the definition starts to include the known risk factors, including but not limited to LDL-C, hypertension, hypertriglyceridemia (HTG), risk factors regarding lifestyle such as inactivity, diet, and smoking, and risk factors for CVD due to comorbidities, like obesity, diabetes, metabolic syndrome (MetS), chronic kidney disease (CKD) and hypertension. The definition of residual CVD risk provided by the Residual Risk Reduction Initiative (R3i) (3) is: *Residual cardiovascular risk is defined as the risk of cardiovascular events that persists in people despite achievements of treatment goals for low-density lipoprotein (LDL) cholesterol, blood pressure, and glycaemia according to current standards of care* (4). The definition we use for residual CVD risk is *the risk of CVD of an individual who is given proper therapy for hyperlipidemia, hypertension, hyperglycemia, and advice for healthy lifestyle, and who is checked for proinflammatory and procoagulant factors*.

## 2. Methods

We reviewed the papers published on residual CVD risk; other topics, such as residual lesions, residual obstructions, residual plaque burden after stenting, residual confounding (in statistics), residual enzyme activities, and residual lipolysis were excluded from this review. A literature search was conducted to identify all published studies that mentioned cardiovascular (CV) residual risk in title, abstract and key words. PubMed databases were systematically searched with the aid of an experienced librarian. The search strategy included the following terms or derivatives of these terms: residual CVD risk, residual thrombotic risk, residual vascular risk, residual atherosclerotic risk, and residual inflammatory risk. We excluded studies involving animals and those without available text in Dutch, German, French, or the English language. Data was collected up to January 12th, 2023, resulting in 989 hits. Titles and abstracts were manually reviewed on (1) the definition of the term residual CVD risk, and (2) the medical, biochemical and/or pathological risk factors involved. We summarized all therapies to reduce residual risk described in this review in Supplementary Table S1. To complete the table with the most recent data, we refer to literature that does not necessarily include residual risk in its title or abstract.

## 3. Residual risk factors

In an early European survey of CVD risk factors and their specific therapies to achieve target levels, treated dyslipidemic patients attained the targets of total cholesterol (TC) (<4.91 mmol/L) and of LDL-C (<2.97 mmol/L) in 42.2%, treated hypertensives attained targets of blood pressure (<140/90 mmHg) in 38.8%, treated type 2 diabetic (T2DM) patients attained targets of hemoglobin-A1c (HbA1c) (<47.5 mmol/mol) in 36.7%, and treated obese patients attained targets of body mass index (BMI) (<30 kg/m^2^) in 24.7% (5). These results clearly indicate that even in patients on treatment roughly half were off target, and at high remaining CVD risk.

### 3.1. Residual risk associated with atherogenic dyslipidemia

#### 3.1.1. LDL-cholesterol

TC and LDL-C were the first lipids identified as responsible for atherosclerotic CVD (ASCVD). Initially, TC and LDL-C were not widely accepted as a risk factor, but statin studies that reduced TC and LDL-C levels supported the lipid theory of atherosclerosis (Supplementary Table S1). Today, LDL-C remains the primary therapeutic target for ASCVD management and prevention, but should not be the only one. Statins are LDL-C lowering drugs that inhibit hepatic cholesterol synthesis through inhibition of hydroxymethylglutaryl-CoA (HMG-CoA) reductase (6). In the statin-treated patient the LDL-C targets should be met. The significant residual CVD risk observed in ≈70% of patients under optimal statin therapy warrants the exploration and testing of alternative risk factors and specific drugs (2). In addition to statins, ezetimibe a cholesterol absorption blocker, monoclonal antibodies alirocumab and evolocumab, which are proprotein convertase subtilisin/kexin type 9 (PCSK9) inhibitors, and inclisiran, a small interfering RNA (siRNA) therapeutic agent that inhibits synthesis of PCSK9, have been reported to effectively lower LDL-C.

##### 3.1.1.1. Small dense LDL (sdLDL)

Under conditions of atherogenic dyslipidemia, a fraction of LDL is apparent with small dense LDL (sdLDL) particles, generally in combination with low levels of HDL-C, and elevated levels of TG, TG-rich lipoproteins (TGRLs) and their remnants. These particles are highly atherogenic and their cholesterol content is considered useful for additional risk stratification and determination of residual CVD risk (7). One year later, the same group reported that sdLDL, HDL-TG and large concentrations of LDL particles were the most powerful predictors of CVD risk (8). Likewise, in patients with acute coronary syndrome (ACS) who underwent percutaneous coronary intervention (PCI) those with elevated levels of sdLDL have higher risk of CV events compared to those without elevated sdLDL levels (9).

##### 3.1.1.2. Apolipoprotein B (apoB)

Apolipoprotein B (apoB) is present in all atherogenic lipoproteins contributing to CV risk: lipoprotein(a) [Lp(a)], LDL, very low-density lipoprotein (VLDL), sdLDL, and chylomicrons (CMs). Therefore, apoB concentration is a direct measure of atherogenic lipoprotein particles’ number in circulation and a more suitable measurand than the LDL-C concentration, which does not directly reflect the total number of atherogenic lipoprotein particles. To our knowledge Fruchart et al. were the first to mention apoB in relation to residual CVD risk in the R3I in which the authors call for action to reduce CV risk despite achieving target levels of LDL-C, blood pressure and glycemia (10). As Sniderman stated in 2009: “*ApoB is simply a better way of measuring LDL-C”* and “*should be the primary target of LDL lowering therapy and not simply measured after cholesterol targets have been achieved*” (11). Indeed, studies have repeatedly shown that apoB outperforms both LDL-C and non-HDL-C as CVD risk predictor in both men and women at all ages (12–16). For example, in the INTERHEART study apoB showed to be a better predictor of myocardial infarction (MI) than LDL-C and non-HDL-C (13). Especially in T2DM patients apoB is an important marker, as CV risk in these patients is related to elevated TGRL levels rather than high LDL-C. In addition, in T2DM patients with elevated TG levels, the Friedewald equation to calculate LDL-C fails. In 2011, a meta-analysis was conducted on apoB as CV risk marker in statin trials, which demonstrated that apoB outperforms LDL-C in CV risk prediction (13). The author concluded that in future guidelines of lipid-lowering therapies, apoB should be mentioned as (1) an indicator of CV risk, (2) an indicator treatment efficacy, and (3) a target of therapy (13). As previously stated, measuring apoB is a more comprehensive way of assessing the total number of atherogenic particles compared to LDL-C. For instance, in the presence of elevated levels of sdLDL, only measuring apoB will provide an accurate picture of the risk of CVD. Relying solely on LDL-C may miss the presence of sdLDL and underestimate the risk of CVD (17, 18).

##### 3.1.1.3. Direct apoB targeted therapy

Mipomersen is an antisense oligonucleotide (ASO) directed at apoB100, preventing the hepatic synthesis of apoB and formation of VLDL and LDL. Mipomersen decreased apoB levels by 36% in patients with severe hypercholesterolemia and in patients with increased CVD risk (19). In mild dyslipidemic patients, mipomersen administration resulted in up to 50% decrease of apoB levels (20). In patients with heterozygous familial hypercholesterolemia (heFH), apoB was decreased by 33%. Homozygous FH (hoFH) patients lacking functional LDL-receptors, are often unable to reach therapeutic target levels with traditional lipid-lowering therapies such as statins or PCSK9 inhibitors that upregulate LDL receptors. Mipomersen administration was able to reduce apoB in hoFH patients already on lipid-lowering therapies by 24%. Despite promising results, mipomersen was rejected by the European Medicine Agency (EMA) due to risk of liver toxicity, because of hepatic accumulation of TG most likely due to impaired VLDL production (20). In contrast, the United States Food and Drug Administration (FDA) did approve mipomersen as treatment of hoFH patients only. Another way to prevent apoB-containing lipoprotein production and secretion is inhibition of mitochondrial triglyceride transfer protein (MTP) with lomitapide (21). In a phase III trial including hoFH patients, lomitapide was able to reduce apoB and LDL-C levels by 49% and 50%, respectively (22). Because of these results, lomitapide administration to hoFH patients has been approved by the FDA and EMA.

#### 3.1.2. HDL

One of the secondary targets for intervention in individuals treated with statins was HDL-C, as low HDL-C was reported to be a characteristic for atherogenic dyslipidemia. Many studies were devoted to therapies that reduced residual CVD risk by increasing HDL-C. In dyslipidemic patients with CVD and in patients with dyslipidemia HDL-C levels are generally low, most often in combination with elevated TG levels. Worldwide, much effort has been paid to treat patients, already on statins, with HDL-raising medication. In the ARBITER 2 trial, among patients with CHD and mean levels of HDL-C and TG of 1.03 mmol/L and 1.84 mmol/L, respectively, therapy with nicotinic acid was associated with increase of HDL-C, decrease of TG, and lack of progression of carotid intima-media thickness (IMT), whereas in controls carotid IMT increased over time (23). A cholesterol-ester transport protein (CETP) inhibitor, torcetrapib, added to atorvastatin therapy, produced a dose-dependent increase in HDL-C, as well as an additional decrease in LDL-C (24). Torcetrapib was withdrawn from clinical testing because of serious adverse effects (25, 26). Besides CETP inhibitors, apoA-I mimetics, recombinant HDL, liver X receptor (LXR) agonists and peroxisome proliferator-activated receptors (PPAR) agonists were advocated as HDL-C raising drugs to reduce CVD risk (27). Pöss et al. presented the warning that an increase of HDL-C does not necessarily imply an improvement of the functional properties of HDL (28). Indeed, the JUPITER trial demonstrated that in statin-treated patients with CVD who had low LDL-C levels, low HDL-C was not predictive of residual CVD risk (29). An important conclusion of the ACCORD trial was that extension of statin therapy with fenofibrate yielded no significant ASCVD risk reduction (30). The ILLUMINATE trial found no improvement of torcetrapib on residual CVD risk, which questions the benefit of HDL-raising therapy (31). The AIM-HIGH trial showed no incremental benefit of niacin with statin therapy after 36-months follow-up (32). As the same was true for CETP inhibitors and fibrates, it was suggested that instead of targeting HDL-C levels, the quality of HDL in terms of particle number, shape, size, and composition e.g., apolipoprotein, triglyceride and cholesterol content and HDL’s functionality should be taken into consideration (33–35). HDL is considered atheroprotective, is involved in reverse cholesterol transport, and has anti-inflammatory, anti-thrombotic, anti-oxidative, anti-infectious, and vasodilatory activities (36). High levels of dysfunctional HDL are associated with increased risk of CVD, whereas high levels of functional HDL, enriched in ApoA-I are associated with decreased risk of CVD (35, 37). Besides ApoA-I, other HDL components, such as HDL-associated hydrolases (e.g., paraoxonase-1), certain (lyso)phospholipids, nutrition, smoking, air pollution, and plastic-associated chemicals influence HDL’s functionality (38). In individuals with very low HDL-C, due to rare monogenic dyslipidemia (e.g., Tangier disease, LCAT deficiency, familial hypoalphalipoproteinemia) or due to secondary dyslipidemias, the very low HDL-C levels are associated with (1) increased risk of CVD, (2) comorbidities, such as T2DM, and (3) elevated levels of sdLDL (39).

##### 3.1.2.1. ApoA-I mimetics

Nicholls et al. wondered whether instead of HDL’s cholesterol content, it would be better to study the beneficial effects of HDL’s apolipoprotein A-I (apoA-I) content in dyslipidemic patients (40). ApoA-I is a protein synthesized in the liver and intestine and contributes to the structure of HDL (41). A successful way to increase HDL-C levels is treatment with apoA-I mimetics, resulting in an enhanced reverse cholesterol transport function of HDL. However, the CARAT trial has demonstrated that patients with ACS who received a recombinant wild-type apoA-I (CER-001; 3 mg/kg body weight weekly) lacked any regression of plaque volume compared to placebo (42).

#### 3.1.3. Hypertriglyceridemia (HTG) and TG-rich lipoproteins

In statin-treated individuals residual CVD risk may be due to persistent atherogenic dyslipidemia, which can be defined by high fasting TG levels (≥2.31 mmol/L) and low HDL-C levels (≤1.0 and ≤1.29 mmol/L in men and women, respectively), sdLDL particles, remnant lipoproteins, and postprandial hyperlipidemia. HTG results from hepatic oversecretion and/or hypocatabolism of TGRLs, being VLDL particles and their remnants (43). Atherogenic dyslipidemia is a characteristic often seen in individuals and patients with obesity, T2DM, and MetS (44, 45), and is associated with an increased (by 58%) risk of CVD (46). Often TG elevations are secondary to several conditions, but are primary to syndromes like familial combined hyperlipidemia, type III hyperlipidemia in combination with the apoɛ2/ɛ2 genotype, and familial chylomicronemia syndrome (FCS) (47). In the FMD-J study serum TG levels >100 mg/dl (1.13 mmol/L) in patients undergoing PCI had increased risk of new events compared with those having TG levels <100 mg/dl (1.13 mmol/L) (48). In primary prevention, individuals with TG levels ≥150 mg/dl (1.69 mmol/L) were at lower (by 9%) adjusted risk of death and higher (by 14%) risk of MACE. In secondary prevention patients with TG levels ≥150 mg/dl (1.69 mmol/L) were at lower adjusted risk of death (by 5%), higher (by 4%) risk of MACE, and higher (3%) risk of all-cause hospitalization (49). Mason et al. considered “*TG levels as a potential biomarker of CV risk, but found no evidence that TG lowering itself is an effective strategy for reducing such risk*” (50). Individuals with HTG having low to moderate risk of CVD suffered from subclinical atherosclerosis and vascular inflammation, even in the absence of hypercholesterolemia (51).

##### 3.1.3.1. Fibrates

While lifestyle modification is key to managing patients with HTG (52, 53), fibrates have been advocated as therapy for HTG for a long time. Fibrates such as fenofibrate and gemfibrozil, which modulate the PPARs, decrease TG and increase of HDL-C. Although these drugs decrease TG, their effect on apoB is limited. Fibrates stimulate free fatty acid (FFA) oxidation in the liver, thereby reducing fatty acids available for VLDL synthesis and secretion. Another effect of fenofibrate is stimulation of lipoprotein lipase (LPL) expression, and its inhibition of apoC-III expression in the liver. Thus, the dual mechanism of TG lowering by fibrates is reduced synthesis, and intensified hydrolysis of TGRLs (54).

##### 3.1.3.2. PPAR modulators-α/K-877

In 2014 Fruchart et al. introduced the R3I that had to find out how to treat atherogenic dyslipidemia (10). This R3I group introduced therapy of atherogenic dyslipidemia with selective PPAR-α modulators (SPPARα), such as pemafibrate (55). In 2015, the PPARα/γ agonist, saroglitazar, was reported to be of substantial benefit for patients with atherogenic dyslipidemia and/or diabetes (56), and in 2017 therapy with statin plus K-877 (pemafibrate) was advocated as therapy with a favorable benefit-to-risk ratio (57). The PROMINENT study was performed with pemafibrate in patients with HTG and T2DM and close to/on target LDL-c levels, but was stopped in April 2022 for reasons of futility (55). While pemafibrate successfully decreased TGRLs and their remnants, it led to an opposing outcome of elevated LDL-C and ApoB levels. Basically, pemafibrate was able to increase the conversion of TGRLs, but did not increase the clearance of the resulting atherogenic lipoprotein particles (58), nor did it reduce the levels of sdLDL-C (59). As to the latter finding, it is clear that in diabetics with rigorous control of LDL-C, TG-lowering therapy does not efficiently suppress sdLDL-C levels, which may explain the lack of suppression of ASCVD risk by pemafibrate (59).

##### 3.1.3.3. ω3-fatty acids

Studies investigating the effects of ω3-fatty acids, including docosahexaenoic acid (DHA) and eicosapentaenoic acid (EPA), on TG levels in patients with T2DM and MetS, have often yielded disappointing results with insignificant reductions in TG. However, in 2012 it was demonstrated that ω3-fatty acids had been given at too low doses to affect lipid profiles (60). EPA demonstrated improvement in atherogenic dyslipidemia and blood pressure, supporting its anti-atherosclerotic role, including preventing occurrence of new events (61). Moreover, EPA lowered TG levels and exhibited anti-inflammatory effects (62). Statin-treated patients with HTG showed favorable lipid changes upon switching to icosapent ethyl, a highly purified, stable ethyl ester of EPA (63). The results of the REDUCE-IT study demonstrated that icosapent ethyl decreased TG levels and reduced the risk of the trial’s primary CV endpoint by 25%, although the causal relationship between the two was not proven (64). The FDA approved icosapent ethyl for adults on statin therapy with TG levels ≥150 mg/dl (1.69 mmol/L) and either CVD symptoms or T2DM and at least two additional CVD risk factors (65, 66). Surprisingly, the icosapent ethyl-induced reduction in CV events was not explained by the reduction in TG alone (67–69), but may be related to other pleiotropic effects induced by an increased EPA/arachidonic acid (AA) ratio (70). This EPA/AA ratio is inversely associated with an increased risk of cardiovascular events in patients with CAD (71). Notably, EPA acts as a cardioprotective factor stabilizing plaque by inducing anti-inflammatory response and reducing platelet aggregation. In contrast, AA destabilizes plaque by activating inflammatory responses and promoting platelet activation. Increasing the EPA/AA ratio by icosapent ethyl administration may therefore lead to improved plaque stability, reduced platelet adhesion, and anti-inflammatory factors (72–74) and improved endothelial function (75).

#### 3.1.4. Lipoprotein remnants

In statin-treated individuals, the incidence rate of CV events is reduced by ≈30%. This means that remaining residual risk is effectuated by factors other than LDL-C, the most frequent being TGRLs and Lp(a). Particularly the accumulation of the relatively cholesterol-enriched, incompletely catabolized remnants of CMs and VLDL has become a new target to reduce residual CVD risk (76, 77). Of the VLDL subclasses identified, the smallest remnant subclass was associated with the highest residual risk (78). In literature there are multiple definitions used for “remnants” in relation to lipoprotein particles and their composition. Usually, remnants of TGRLs are referred to as remnant lipoproteins and the cholesterol content of those remnants are reported as remnant cholesterol (RC). However, there is no consensus on the definition of RC as the ways RC are calculated and measured differ widely among studies. Varbo and Nordestgaard referred to RC as non-HDL-C minus LDL-C, which can be referred to as calculated RC (79). This means that it includes the cholesterol content of unmetabolized VLDL, intermediate density lipoprotein (IDL) and CMs (non-fasting) and not just their remnants. Unless specified otherwise, we will be referring to calculated RC when discussing RC.

Remnant lipoproteins are formed through lipolysis of VLDL and CM, resulting in enrichment of cholesterol (both free and esterified) and depletion of TG content. Efficient lipolysis of TG in VLDL particles by LPL results in a rapid conversion to regular-sized LDL, with limited formation of remnants. However, when lipolysis is retarded, more remnants are formed and can accumulate, leading to a prolonged residence time in circulation. On top of that, slower lipolysis leads to the formation of sdLDL. Remnant lipoproteins are either cleared directly via hepatic uptake or converted to IDL and LDL. Multiple factors can impair lipolysis such as VLDL accumulation, elevated apoC-III levels, and lower LPL activity due to mutations.

In 2013, Varbo et al. found that elevated levels of RC is a causal factor for both increased risk for ischemic heart disease (IHD) and low-grade inflammation in the general Danish population (80). In 2016, Jepsen et al. showed in the Copenhagen Ischemic Heart Disease Study that RC in IHD patients was associated with increased risk and all-cause mortality (81). Measured RC was also associated with this increased risk, although less strongly than the calculated RC, including VLDL and IDL cholesterol. Interestingly, this increased risk was not associated with elevated levels of measured LDL-C, suggesting a role for RC in addressing residual all-cause mortality risk for patients with IHD. The authors concluded that 8%–18% of residual risk of all-cause mortality in IHD patients can be attributed to elevated levels of RC (81). In the NHANES study population, Zhang et al. demonstrated that elevated levels of RC were associated with increased risk of CV mortality, independent of HDL-C and LDL-C (82). The authors concluded that the time has come to address residual CVD risk by targeting RC. Fasting plasma apoB48 levels are correlated with severity of CAD (83). Patients with high levels of chylomicron remnants should be managed with anti-diabetes therapy, complemented with a low-fat diet.

##### 3.1.4.1. Remnants and therapies

Multiple approaches exist to target TGRL formation, remnant formation, and elevated RC. ApoB is crucial to particle formation as TGRLs require one apoB molecule per particle. Decreasing TGRLs, their remnants, and RC can be achieved by targeting apoB synthesis. Approaches include inhibiting apoB formation with mipomersen or inhibiting the assembly of VLDL by MTP inhibition with lomitapide. Another approach involves the increase of LPL activity to clear TGRLs. This can be accomplished by inhibiting apoC-III or ANGPTL3 synthesis, which are both lipolysis inhibitors.

###### 3.1.4.1.1. ApoC-III inhibition

ApoC-III acts through various mechanisms: (1) it is an inhibitor of LPL and hepatic lipase (HL), (2) it impairs apoE-mediated hepatic uptake of TGRL, (3) it facilitates VLDL-TG assembly and secretion, (4) it impairs apoB100-mediated binding and apoE-mediated binding of LDLR and LRP-1, resulting in a decreased hepatic uptake of VLDL and CM, and (5) accumulation of apoC-III leads to conformational changes of HDL resulting in decreased apoA-I content, impaired insulin sensitivity and reduced cholesterol efflux capacity (18, 84). ApoC-III was significantly associated with CV events in patients with stable CAD (85). Interestingly, the prognostic value of apoC-III was less strong in the presence of CMs (non-fasting). In addition, individuals with loss-of-function *APOC3* showed 40% lower plasma TG levels, 40% lower risk for CHD, and 60% lower risk for ischemic vascular disease compared to non-carriers, implying a causal relationship between apoC-III and CVD (85, 86).

Today, inhibition of apoC-III expression seems a new, promising target to normalize the concentrations of TG and remnants (81). One of the first anti-apoC-III agents that became available was volanesorsen (formerly ISIS 304801, ISIS-APOCIII-Rx), an ASO, which reduced TG levels by 76.5% and plasma apoC-III levels by 84.2% in patients with familial chylomicronemia syndrome (FCS). The FDA did not approve volanesorsen in FCS patients since a substantial proportion (76%) of these patients developed thrombocytopenia in the APPROACH trial (87). In contrast, the EMA did approve volanesorsen therapy, but in in patients with FCS only (88). Newer apoC-III-antagonists, like the ASO olezarsen (formerly AKCEA-APOCIII-LRx), an N-acetylgalactosamine (GalNAc) conjugated form of volanesorsen, showed apoC-III reduction of 92% and TG reduction of 77% in healthy individuals with mildly elevated TG levels (85). Whether olezarsen improves clinical outcome is yet unknown.

###### 3.1.4.1.2. Angiopoietin-like protein 3 inhibition

ANGPTL3, like apoC-III, acts as a lipolysis inhibitor and presents another target to increase LPL activity. Individuals with loss-of-function *ANGPTL3* had lower levels of plasma TG, LDL-C and HDL-C and lower CV risk compared to non-carriers (89, 90). ANGPTL3 is an inhibitor of LPL, an enzyme responsible for lipolysis of apoB-containing lipoproteins. Novel ANGPTL3 inhibition strategies, such as monoclonal antibodies (evinacumab), ANGPTL3 ASO (IONIS-ANGPTL3-LRx), and siRNA against ANGPTL3 (ARO-ANG3), have in common that they increase the rate of lipolysis and reduce LDL-C and TG levels (87).

#### 3.1.5. Lp(a)

Lp(a) is a lipoprotein containing a plasminogen-like glycoprotein apo(a) covalently bound to an apoB100-containing LDL-like particle. Unlike most other types of lipoproteins, Lp(a) levels are largely determined by genetics and are not significantly affected by lifestyle characteristics such as nutrition and exercise. The precise mechanism by which Lp(a) operates is uncertain, but Lp(a) is thought to contribute to ASCVD via pro-atherogenic, pro-inflammatory, and/or pro-thrombotic pathways. In 2011, Mangalmurti et al. mentioned assessment of Lp(a) level, apoB level and LDL particle number as lipid biomarkers that “*potentially have clinical utility*” (15). The AIM-HIGH trial with patients with previous ASCVD on statin treatment in combination with niacin, showed that Lp(a) was a risk factor for recurrent ASCVD in the group with combination therapy and in the control group (only statins), whereas apoB and apoA-I (corresponding to all atherogenic lipoprotein particles and corresponding to HDL particle number, respectively) were only predictive for recurrent ASCVD in the control group, suggesting an independent role for Lp(a) in relation to ASCVD (91, 92). Indeed, compelling pieces of evidence from clinical trials, such as AIM-HIGH and JUPITER, and meta-analyses have now consistently shown that Lp(a) is a risk factor for atherosclerosis and CVD independent of LDL-C levels (93, 94). Elevated levels of Lp(a) are an independent risk factor for aortic valve stenosis (95). This has been supported by Mendelian randomization studies that suggest a causal relationship between elevated levels of Lp(a) and the occurrence of both ASCVD and aortic stenosis (96). Already in 2016, Tsimikas discussed the role of Lp(a) in primary and secondary prevention of CVD and concluded that one measurement of Lp(a) can reclassify 40% of the patients in intermediate risk score categories in primary care (92). Averna and Stroes together with the Expert Working Group on Lipid Alterations Beyond LDL conducted a thorough evaluation of clinical data resulting in recommendations addressing residual CVD risk with biomarkers beyond LDL-C, such as non-HDL-C, apoB, RC and Lp(a). The authors state that Lp(a) is a strong, genetic, independent, and causal risk factor for CVD and should be considered measuring in patients with premature CVD, FH, and family history of CVD (97). The recent identification of a correlation between Lp(a) level and CVD risk has resulted in updated guidelines that suggest Lp(a) measurement in specific clinical situations. In 2020, Tsimikas et al. conducted a meta-analysis including twelve statin trials and concluded that statins significantly increase Lp(a) from baseline up to 24.2% and stressed the importance of investigating the Lp(a)-attributable residual CVD risk after statin treatment (98).

##### 3.1.5.1. Lp(a) and oxidized phosholipids (OxPL)

The OxPL components of Lp(a) are proinflammatory and contribute to proatherogenic properties of Lp(a). Lp(a) is the primary carrier of plasma OxPL (about 85%), even though the number of Lp(a) particles is considerably lower than that of LDL (99). Several studies showed that OxPL-apoB is equivalent or superior to Lp(a) as a marker for diagnosis and prognosis of CVD and calcific aortic valve stenosis (100). OxPL on Lp(a) has also been shown to up-regulate genes related to inflammation (100). In addition, OxPL-apoB levels were elevated in patients with ACS or ASCVD and were highly predictive for the risk of MI, stroke and CV mortality (112). As statins are known to increase Lp(a) levels, statins may thus also increase OxPL-apoB levels. Simvastatin/ezetimibe administration led to a mean increase in OxPL-apoB of 24% and an Lp(a) increase of 11%. The ASO directed at apo(a), pelacarsen, was able to reduce OxPL levels, besides Lp(a) (100).

### 3.2. Residual risk associated with inflammatory processes and factors

Recent studies have confirmed that inflammation increases CV risk independent of LDL-C levels. Especially atherosclerosis is now widely accepted as a chronic low-grade inflammatory condition, in part caused by cholesterol itself (101). Several biomarkers of inflammation have been studied in relation to atherosclerosis and subsequent plaque formation. Here we discuss the central inflammatory signaling pathway and phospholipases as targets to address residual inflammatory risk.

#### 3.2.1. IL-1-to-IL-6-to-CRP signaling pathway

High sensitivity C-reactive protein (hsCRP) provides the most substantial evidence as a useful prognostic inflammatory marker for residual inflammatory risk in patients at target levels of LDL-C. Despite hsCRP being a valuable marker for increased risk (102), research on the association between genetic variants in the CRP gene and CHD risk suggest that CRP is not likely a causal factor in CHD (103). Instead, a number of Mendelian randomization studies found causal relations between the IL-6 receptor gene and the risk for CHD (104). The JUPITER trial was the first significant clinical study that examined whether CRP could be used as novel biomarker to identify patients who could benefit from statin therapy, but who were on target LDL-C levels and therefore not eligible for lipid-lowering according to guidelines (105). This trial showed that patients with LDL-C levels <130 mg/dl (3.36 mmol/L) and CRP levels of ≥2 mg/L had a higher risk of CV events compared to patients with low LDL-C and CRP <2 mg/L. Moreover, CRP was found to be a stronger predictor of these events than LDL-C (106, 107). Assessing both LDL-C and CRP levels together provided superior prognostic information than testing for either measure alone (120). This was supported by other clinical trials (SATURN, PROVE-IT, AFCAPS/TexCAPS, REVERSAL, and IMPROVE-IT) (108–110).

CANTOS was the first clinical trial that directly investigated the relationship between atherothrombosis and inflammation regardless of lipid levels. Canakinumab, an IL-1β antagonist, directly inhibits IL-1-to-IL-6-to-CRP signaling pathway. In 2017, CANTOS showed a 26% reduction of MACE for patients with on-treatment hsCRP <2 mg/L after canakinumab administration, independent of LDL-C lowering, compared to the placebo group (111). In addition, in this subgroup CV mortality and all-cause mortality were significantly reduced by 31%. However, canakinumabs’ clinical applicability is hampered due to high prevalence (over 10%) of adverse events including neutropenia, cellulitis, pseudomembranous colitis, fatal infection, and sepsis, as well as expensive treatment costs (112).

In parallel with CANTOS, the CIRT with methotrexate was conducted. Initially, methotrexate was a chemotherapeutic drug acting as a folic acid antagonist, and it is commonly used to treat rheumatoid arthritis and psoriasis. A cross-sectional study involving rheumatoid arthritis patients revealed that methotrexate was associated with a 15% reduction in CV events, indicating its potential as a promising new therapeutic approach for CVD (113, 114). However, when tested in a CV context, methotrexate did not reduce levels of IL-1β, IL-6 or CRP among patients with stable atherosclerosis, nor did it lead to a reduction in CV events compared to placebo (115).

Colchicine, another anti-inflammatory agent, inhibits NLRP3 inflammasome activation and the downstream activation of IL-1, IL-18, and IL-6. The COLCOT trial showed a 23% risk reduction in MACE with colchicine administration after MI (116). In the LoDoCo2 trial, colchicine administration to stable CAD patients resulted in a 30% reduction in CV events compared to placebo. However, it can cause myalgia, gastrointestinal distress, and drug interactions with commonly prescribed medications, including antibiotics and statins (117).

Bempedoic acid is a therapeutic agent that inhibits ATP citrate lysase, just upstream from HMG-CoA reductase, lowers LDL-C, and reduces hsCRP. The CLEAR Outcomes trial assessed its effects in patients with ASCVD or heFH on statin therapy with residual inflammatory risk (hsCRP ≥2 mg/L). Results show that bempedoic acid lowers lipid levels (LDL-C, TC, and apoB) and inflammation (IL-6 and hsCRP) independently, making it a promising candidate for residual cholesterol-related and inflammatory risk. However, it has no impact on Lp(a) levels (118).

Recently, ziltivekimab, an IL-6 inhibitor, has shown to effectively reduce hsCRP up to 92% in individuals with elevated hsCRP and CKD (119). Currently, the ZEUS trial is investigating its impact on reducing hsCRP and MACE.

#### 3.2.2. Lipoprotein-bound phospholipase A2 (Lp-PLA2)

PLA2 is a family of enzymes that is responsible for the hydrolysis of oxidized phospholipids on LDL particles, resulting in the production of two highly inflammatory mediators, lysophosphatidylcholine and oxidized FAs, which can be linked to atherosclerotic plaque formation and plaque inflammation (120).

In 2005, Lp-PLA2 was a novel inflammatory marker of CV risk that was being considered as a potential therapeutic target (121, 122). Lp-PLA2 is primarily bound to LDL, but also to HDL, Lp(a) and TGRLs. Multiple studies have shown that elevated levels of Lp-PLA2 are associated with increased risk of CHD and stroke, independently of hsCRP and after adjusting for traditional risk factors (105). Lp-PLA2 seemed to be an interesting target as it is a cross-over between lipid metabolism and inflammation, both involved in CVD risk. In 2008, a phase II trial was conducted with darapladib, an Lp-PLA2 inhibitor, in patients with CHD. Darapladib reduced interleukin-6 (IL-6) and hsCRP levels and prevented necrotic core expansion in coronary atherosclerotic lesions (123). However, in two trials darapladib administration in patients with recent ACS and in patients with stable CHD did not lead to a reduction in MACE (SOLID-TIMI 52 and STABILITY). This implies that Lp-PLA2 may be a biomarker for vascular inflammation instead of being a direct cause of CVD. In addition, darapladib administration led to adverse side effects such as diarrhea and malodorous feces, urine, and skin (123). Notably, patients in both trials had low levels of LDL-C and the majority was taking statins, which inhibits PLA2 activity. These findings suggest that targeted PLA2 inhibition on top of statin treatment does not offer any additional benefit (124).

#### 3.2.3. Endothelial dysfunction

Endothelial dysfunction is a general term that describes the site that attracts, binds, and internalizes monocytes that may develop into foam cells and subsequent plaque formation. Besides, dysfunctional endothelium produces less nitric oxide (NO), a vasodilator, due to depressed eNOS (NOS3) activity. Instead, in dysfunctional endothelium inducible NOS (iNOS or NOS2) is formed, ultimately leading to massive quantities of peroxynitrite, a molecule with detrimental effects on tissues, such as hypertrophy, dilatation, fibrosis, and dysfunction. Factors that contribute to endothelial dysfunction include dyslipidemia, oxidative stress, and inflammation (125). Statins have been reported to improve endothelial dysfunction (126). HTG was recognized as a therapeutic target in the treatment of endothelial dysfunction and *ω*3-fatty acids administration was recommended as therapy to improve endothelial function (61, 127). When patients with CAD and impaired vascular function underwent optimal medical treatment for 24 weeks, the improvements of flow-mediated vascular dilatation, a marker of vascular endothelial function, predicted the lowest probability of future MACE (128).

#### 3.2.4. Clonal hematopoiesis of indeterminate potential (CHIP)

Clonal hematopoiesis of indeterminate potential (CHIP), a collection of somatic mutations, is an age-associated risk factor for MI, stroke, heart failure events, and survival following percutaneous aortic valve intervention (129, 130). It is suggested that CHIP activates the inflammasome pathway and contributes to thrombosis, leading to CVD. Although the association between CHIP and CVD is still being studied, early evidence indicates that CHIP may serve as a useful biomarker for identifying those at increased risk of CVD (129, 130). There are no specific therapies yet.

### 3.3. Residual risk associated with thrombotic processes and coagulation factors

Current guidelines to reduce atherothrombotic events involve antiplatelet therapy and lipid-lowering therapy. However, a residual risk of atherothrombosis and subsequent CV events remains in secondary prevention of CVD after coronary intervention (131, 132). There are currently two commonly used therapeutic approaches to address residual thrombotic risk, namely dual antiplatelet therapy (DAPT) and dual pathway inhibition (DPI).

#### 3.3.1. Dual antiplatelet therapy (DAPT)

The platelet P2Y12 receptor has a key role in thrombus formation during ACS. Dual antiplatelet therapy, combining aspirin and a P2Y12 inhibitor, was used to decrease residual thrombotic risk, at the expense of a bleeding risk. The PEGASUS-TIMI 54 trial with ACS patients with stable CAD demonstrated that treatment with P2Y12 inhibitor, ticagrelor, on top of aspirin administration resulted in a 16% reduction in MACE (132, 133). Particularly after invasive procedures, dual antiplatelet therapy proved to be highly effective in preventing thrombotic events. Also, in diabetics anti-thrombotic strategies in acute and chronic CAD remain an unmet clinical need (134).

#### 3.3.2. Dual pathway inhibition (DPI)

A relatively novel approach to address this residual thrombotic risk is dual pathway inhibition (DPI). DPI involves targeting both platelet activation and coagulation cascade by combining antiplatelet and anticoagulant agents. The COMPASS trial with patients with stable ASCVD showed that the combination of rivaroxaban (a Factor Xa inhibitor) and aspirin was superior in preventing recurrent MACE compared to aspirin alone, but at the expense of significant bleeding risk (135). Low-dose rivaroxaban in combination with aspirin has been implemented in European guidelines for patients with diabetes and peripheral artery disease at low bleeding risk (136). The combination of rivaroxaban on top of clopidogrel has been examined in patients with ACS, resulting in significant reduction of ischemic events and CV mortality, again at the expense of increased risk of bleeding (137, 138).

### 3.4. Comorbidities

Even after controlling for the traditional CV risk factors, subjects with T2DM, MetS, hypertension, obesity, and/or CKD remain at high residual CVD risk despite target LDL-C levels.

#### 3.4.1. Diabetes

DM is one of the comorbidities associated with considerable residual risk of CVD. Already in 2001, diabetes, smoking, hypercholesterolemia, and hypertension were mentioned as correctable risk factors that should be addressed by physicians “*before cardiovascular and renal damage become manifest*” (139). In patients with T2DM, hyperglycemia and dyslipidemia were found to be associated with inflammatory risk, thrombotic risk, and risk of endothelial dysfunction (140). In the following years, many papers reported on new therapies to treat diabetic dyslipidemia, characterized by elevated levels of TG, reduced levels of HDL-C, elevated levels of remnant lipoproteins, and presence of sdLDL. The addition of fibrates to anti-diabetic therapy improved lipid abnormalities, reduced progression of atherosclerosis, and reduced risk of CAD in T2DM patients (44). Fibrates, being agonists of PPAR, can treat insulin resistance and HTG when combined with improvement of diet and physical activity (141). Particularly PPAR-*γ* activators, such as the thiazolidinediones, have, in combination with statins, complementary effects on CVD risk reduction in patients with T2DM (142). Fenofibrate was shown to offer macrovascular and microvascular benefits in patients with T2DM on statin therapy (143–145). However, the FIELD study demonstrated that fenofibrate did not reduce MACE in patients with T2DM, although fenofibrate was shown to have favorable impact on a number of nonlipid residual risk factors (146). Around 2010 it became evident that the ACCORD trial has demonstrated that in patients with T2DM with atherogenic dyslipidemia the combination therapy of statin and fibrate resulted in risk reduction, although in the absence of atherogenic dyslipidemia this favorable effect was absent (147–149). In patients with diabetes or MetS who achieved their desirable LDL-C levels, non-HDL-C levels may remain too high, and deserved specific therapy to reduce residual CVD risk (150). In the year 2012, it became clear that atherogenic dyslipidemia in patients with T2DM or MetS should not be treated with fenofibrate, torcetrapib or niacin in combination with statin to reduce residual risk. Instead, several authors recommended that correction of hyperglycemia should be combined with statin and lifestyle changes (151, 152). Around 2012 several reports proposed that therapy with *ω*3-fatty acids may treat HTG and may reduce residual risk in T2DM patients and patients with MetS (152, 153). By using lifestyle changes, anti-glycemic agents, and lipid-regulating therapies in patients with T2DM, endothelial function improved as well (125). Recently it was found that statins could slightly increase the risk of T2DM, but T2DM patients clearly benefit from statin therapy (154). In the REDUCE-IT trial icosapent ethyl markedly lowered residual risk of MACE in patients with ASCVD and with T2DM (155). Xiao et al. pointed out that the central abnormality of the atherogenic dyslipidemia in diabetics is the presence of TGRLs (remnants) that are primarily responsible for high residual risk (156). From 2019 on, several groups stated that HTG in diabetics is a serious risk factor that deserves therapy (157–161). As diabetes is a leading cause of CKD, new therapies with glucagon-like peptide-1 receptor agonists (GLP-1-RA) and sodium/glucose cotransporter 2 inhibitors (SGLT2i) showed antihyperglycemic effect, and reduced all-cause mortality and CV mortality. GLP-1-RA had favorable effects on diabetic nephropathy (162). SGLT2i have, besides glucose-lowering action, renoprotective effects in diabetics, thereby reducing the rates of end-stage kidney disease and acute kidney injury (AKI) (163–165), and promoting cardioprotection in diabetics (165, 166).

#### 3.4.2 Metabolic syndrome

MetS is a complex condition with metabolic risk factors, like abdominal obesity, atherogenic dyslipidemia, high blood pressure, high plasma glucose, and a combination of prothrombotic and proinflammatory factors. Patients with MetS have high risk of ASCVD and predominant risk factors are abdominal obesity and diabetes. Therapy includes a combination of treatments for high LDL-C, high blood pressure, and diabetes, and includes improvement of lifestyle (167). Treatment of the atherogenic dyslipidemia in patients with MetS reduces residual CVD risk that remains with a statin. The authors advocated the use of fenofibrate that showed a 27% relative risk reduction for CV events (168). To prevent CV and renal events in patients with MetS the newer glucose-lowering medications, SGLT2i and GLP-1-RA, were recommended (169).

#### 3.4.3. Hypertension

Anti-hypertensive therapy, even if blood pressure is at target, has its own contribution in CVD risk, as hypertensives on treatment had a higher risk of stroke than untreated individuals with normal blood pressure (170). But how low should the blood pressure be in treated hypertensive patients? Yannoutsos et al. stated that “*the concept of ‘the lower the better’ tends to be abandoned*”. But in 2010, the “*J-curve concept*” was still the subject of many studies and controversies (171). In a subanalysis of the PRIME trial, which involved patients treated with anti-hypertensive agents or lipid-lowering agents, anti-hypertensive therapy at baseline was significantly associated with risk of CV events, after adjusting for classic risk factors. It was concluded that patients who were treated for hypertension had “*sizable residual cardiovascular risk*”, and deserved more efficient risk reduction (172–174). The range of success of anti-hypertensive therapy, studied in a multi-country survey, was 32.1–47.5%, suggesting that “*efficient risk reduction*” is an effort to work on (5). Apparently, treatment of hypertension cannot completely reverse the sustained vascular damage (e.g., arterial stiffness) as well as other CV morbid conditions (e.g., left ventricular hypertrophy). This extra high residual CVD risk is best predicted by BNP and its inactive fragment NT-proBNP. (175) Even in treated hypertensive patients roughly 30% suffer from left ventricular hypertrophy (29%), diastolic (21%) or systolic (6%) ventricular dysfunction, left atrial expansion (15%), and silent myocardial ischemia (6%). In 13% of this group three or more of these abnormalities occur in combination (176). Apparently, anti-hypertensive therapy does not alter all components of the underlying mechanisms of hypertension that confer CV risk independent of blood pressure (176). Current guidelines therefore advise lifestyle changes, lipid-lowering therapy, antiplatelet therapy and fasting glucose management depending on the risk profile of the patient (177).

#### 3.4.4. Obesity

There are several mechanisms by which obesity can increase the risk of CVD as it is associated with an increased risk of hypertension, dyslipidemia, insulin resistance, T2DM, inflammation, and oxidative stress (178, 179). Already in 2007, Ryan et al. suggested the use of waist circumference (WC) as a measure of abdominal obesity, instead of BMI (180). Indeed, Dhaliwal et al. showed in a cohort study with subjects with no previous diabetes, heart attack, or stroke that WC and waist-to-hip ratio (WHR) both independently predicted CV deaths, whereas BMI did not have any predictive value (181). CRP, marker of inflammation, did not differ between the group that experienced CVD and the group without CVD, suggesting that WC is independently associated with CVD regardless of inflammation (182). However, abdominal obesity was associated with inflammation since adipose tissue was considered to generate inflammatory cytokines leading to a higher inflammatory profile in obese individuals (183). The case-control INTERHEART study demonstrated that the population attributable risk (PAR) of acute MI was greater for abdominal obesity than for diabetes or hypertension (183). Currently, bariatric surgery is one of the most effective interventions to reduce obesity. New drugs to treat obesity and reduce risk of MACE are semaglutide and tirzepatide (184–186).

#### 3.4.5 Chronic kidney disease

Individuals with CKD have a greater risk of CVD compared with the general population but have largely been excluded from clinical trials. CKD patients on dialysis show little to no CV benefit from lipid-lowering therapy and thus have exaggerated residual CVD risk. Probably some of the residual risk in CKD patients is explained by changes in the level, composition, and functionality of HDL, which may contribute to the excess risk of CVD (187). Thus, therapy should be aimed at improving HDL function, possibly by targeting specific moieties within the HDL particle (188). In patients with CKD elevated levels of VLDL-C and apoB, and low levels of HDL-C and apoA-I, are associated with increased risk of ASCVD (189). In patients with combined CKD and atherosclerosis, inflammation is a major predictor of residual CVD risk. Interestingly, in this CKD group elevated levels of LDL-C were not associated with MACE and all-cause mortality (190).

### 3.5. Lifestyle behavior

Healthy lifestyle measures are important to recommend to patients with residual CVD risk, particularly when there is hypertension, obesity, hyperlipidemia, and diabetes. The effectiveness of imposed modifications of lifestyle measures should be assessed regularly, including blood pressure, LDL-C, BMI (and/or WC), and plasma glucose. Physical activity, healthy nutrition, tobacco cessation, alcohol moderation, stress reduction, and weight loss play an important role in the management of the patient with residual CHD, including prevention of new CV events.

#### 3.5.1. Diet

Chronic overnutrition and consequential visceral obesity is associated with a cluster of risk factors for CVD and T2DM (191). The PREDIMED study has demonstrated a 30% reduction in the risk of onset of CVD in patients allocated to a Mediterranean diet as compared to patients with a low-fat diet. Amar et al. suggested that the impact of the probiotic Mediterranean diet on the microbiome causes the beneficial effects on CVD risk and should be considered in prevention of CVD (192, 193). The effect of diet on the microbiome in relationship to CVD has gained more interest over the years. Future research on microbially produced metabolites may lead to new ways to improve CV health (194). Intermittent fasting and variations of it, such as the fasting-mimicking diet, have been linked to improvements in CVD risk markers, including BMI, blood pressure, cholesterol, TG, and CRP (193).

#### 3.5.2. Inactivity

Although the beneficial effects of physical activity on primary and secondary prevention of CVD have been well known for decades, exercise recommendations by clinicians are generally not followed at all or only followed for a brief time. Physical activity has been shown to significantly increase HDL concentration and HDL particle size as well as decrease sdLDL, LDL-C, VLDL and TG (193, 195). In addition, physical activity correlates with lower incidence of CVD up to 50% (195). Furthermore, an inverse relationship between the frequency and intensity of physical activity and all-cause mortality has been observed (195). Combination between caloric restrictive diet and physical exercise demonstrated an independent role for physical exercise in lowering LDL particle number and increasing LDL particle size and HDL particle size (196).

#### 3.5.3. Smoking

In the INTERHEART study, a case-control study that examined the contribution of various cardiometabolic risk factors to the risk of AMI, showed that the PAR of AMI was greatest for dyslipidemia and smoking (183). Another study that estimated the benefit of meeting guideline-recommended targets showed that smoking cessation in a group of 55 patients with acute ischemic stroke led to an absolute 10-year risk reduction of 14% with a median increase of 3.4 CVD-free life years (197).

### 3.6. Unmodifiable risk factors or risks

Unmodifiable risk factors such as age, gender, and genetics contribute to the overall CV risk. Despite advances in medical treatment and lifestyle modification, the remaining residual CVD risk highlights the need for ongoing research to explore new strategies.

#### 3.6.1. Women’s burden of CVD

Women have often been neglected in medical research and have not received adequate representation, recognition, diagnosis, or treatment in various fields, including cardiology.

##### 3.6.1.1. Understudied

The underdiagnosis of CVD in women is partially due to their underrepresentation in clinical trials. This is due to several factors, including an older age of presentation of CVD than men. Thus, women may not meet the age requirements for clinical trials, and women may have a higher reluctance to participate (198). This has resulted in a significant gender imbalance in clinical trial populations. For instance, recent trials such as ODYSSEY Outcomes, FOURIER, and IMPROVE-IT, which focused on dyslipidemic patients with CVD, had a study population that was only 25% female, despite women accounting for 49% of the clinical hyperlipidemic population (199).

##### 3.6.1.2. Underrecognized

Women have both biological risk factors specific to their sex and additional risk factors related to their gender that increase the risk of CVD. Risk factors related to stress, such as depression, poor socioeconomic status, and partner violence, are more prevalent in women and result in an increased CVD risk (198). For example, depression is two-fold more prevalent in women than in men and is strongly associated with IHD (200). Biologically driven risk factors specific to women such as preterm delivery, gestational diabetes, gestational hypertension, premature menopause, and polycystic ovary syndrome (PCOS) are all risk factors contributing to the CV burden in women (201). In addition, the predictive value of CVD risk factors differs between women and men. For instance, hypertension and diabetes have a stronger predictive value for CAD risk in women than in men (200).

##### 3.6.1.3. Underdiagnosed

Specific risk factors contribute to underdiagnosis of CVD in women. For example, the different presentation of ACS symptoms leads to a higher risk of death compared to patients who experience chest pain. This atypical presentation of symptoms for ACS is more prevalent in women than in men, resulting in a higher mortality rate in women. On top of that, even when both men and women show no symptoms of chest pain, women still face a higher mortality risk compared to men in similar circumstances (200).

##### 3.6.1.4. Undertreated

Once women have been diagnosed with CVD, they are less likely to receive appropriate treatment according to clinical guidelines. For example, they are prescribed a lower dose of medical therapy compared to that recommended by guidelines (200). The disparity between women and men in terms of CV health outcome is caused by a combination of factors, including presenting CVD at an older age, longer pre-hospital delays, lower rates of guideline adherence, socioeconomic and cultural disadvantages, and biological differences specific to women.

#### 3.6.2. Age

Berry et al. conducted a meta-analysis using data from eighteen cohort studies involving black and white men and women whose risk factors for CVD were measured at the ages of 45, 55, 65, and 75 years. They observed marked differences in the lifetime risks of CVD across risk factor strata, and whatever the risk factor the risk of each group was evidently dependent of age (202).

#### 3.6.3. Ethnicity

The risk of developing CVD varies among different ethnicities, with the highest risk being found in individuals of sub-Saharan African, Chinese, and Southeast Asian descent (203). Well-established risk factors mostly reflect this increased risk in these populations, such as lower HDL-C levels and higher TG levels in South Asians. In contrast, the levels of LDL-C and apoB are similar across different ethnic groups. However, OxPL-apoB is more prevalent in African Americans than in Caucasians or Hispanics (12). Lp(a) shows the greatest variability between ethnic groups, with African descendants having twice the levels of Lp(a) compared to Caucasians. Interestingly, elevated levels of Lp(a) are not associated with subclinical calcific aortic valve disease in Hispanics, or Chinese individuals, while this association is seen in individuals of European and African descents (203).

## 4. Discussion

We described a variety of risk factors contributing to residual CVD risk. It is evident that it is impossible to treat all these risk factors in every individual to limit residual CVD risk. Current clinical practice operates in a fragmented way, with sometimes too little interaction between clinicians, general practitioners, dieticians, and the patient involved. The transition towards the implementation of pro-active P5 medicine, which encompasses Predictive, Preventive, Personalized, Participatory, and Psycho-cognitive approaches, should be the optimal course of action at population and individual level (204, 205). Instead of a “*one-size-fits-all*” approach, healthcare is slowly moving towards a personalized medicine strategy (206). Thus, to determine the individual’s risk of CVD, one needs biologically meaningful biomarkers that describe the (patho)physiological state of that individual in terms of CV risk prediction. For example, traditional lipid parameters are not refined enough to describe the lipid metabolic state of an individual. Apolipoproteins are emerging biologically meaningful biomarkers that show a more refined picture of the different mechanisms involved in lipid metabolism of an individual than traditional lipids. For example, apoB is part of all atherogenic lipoproteins and its concentration in serum is superior to that of LDL-C and non-HDL-C in predicting CV events in ACS patients (207). ApoA-I is part of, but not restricted to, HDL, and apoC-I, apoC-II, and apoC-III in VLDL, IDL and remnant lipoproteins tend to regulate delipidation of several lipoproteins. Also, Ruhaak et al. reported in 2019 that “*measurement of apolipoproteins in atherogenic particles is more biologically meaningful than the measurement of the cholesterol concentration contained in these particles*” (18). We need to move towards an overall health profile to predict the CV risk of a patient rather than looking at individual markers. The current lipid panel is too limited to capture the full complexity of lipid metabolism in patients with dyslipidemia (17). Prevention is undoubtedly the most effective strategy for the individual’s health, as well as for mitigating the escalating costs of healthcare by avoiding expensive interventions. In today’s practice, however, we notice that T2DM and obesity are gaining widespread prevalence, and that the ban on smoking is circumvented by the popularity of the e-cigarette. At ages older than 50 years the practice of exercise is becoming increasingly sparse. What we need is a more refined approach, including biomarkers, lifestyle advice, family history of CVD, and treatment of comorbidity, that allows personalized medical decision making based on individual patient characteristics. Attention to gain consciousness of the patient regarding his/her own health care is essential. For example, the patient should take responsibility in respect of adherence to primary and secondary prevention, including lifestyle improvements, as advised by clinicians and other caretakers. However, the patient should be supported in this. For instance, governments should devise policies to mitigate the current obesity epidemic and some efforts have shown promising results (208, 209). Psycho-cognitive factors come into play as well, as each patient is unique, not only in terms of biology, but also regarding habits, behaviors, personality, and cognitive dispositions. The patient should undergo a transition from passive bystander to engaged stakeholder, being actively involved in the clinical decision making regarding his/her own health. One of the worrying factors here is the fact that although each person is to be treated on an individual basis, the therapy guidelines are created on the basis of average results from RCTs compiled from groups.

**Figure 1:**
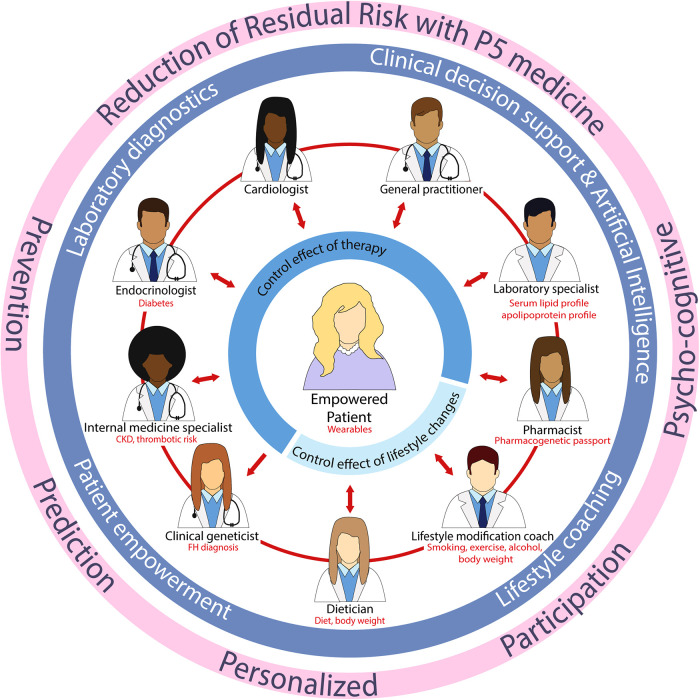
Future clinical practice with the patient centralized and an integral approach between healthcare professionals. CKD, chronic kidney disease; FH, familial hypercholesterolemia.

Digital medicine can aid to move forward towards P5 medicine, in terms of prediction through the collection of big data and artificial intelligence (AI), prevention through monitoring of patient characteristics, and personalized and participatory by involving the individual patient when carrying wearable devices, for example. In our view, digital medicine can be helpful in early diagnosis and monitoring of CVD and thereby reducing residual CVD risk. For example, digital wearables like commercially available smartwatches are already useful in CV risk assessment, prevention, diagnosis, and management (210, 211). As mentioned before, adherence to lifestyle changes has been reported to be challenging. Wearables can be of great value in this area as they can monitor inactivity and give motivational targeted feedback, placing the patient in the lead of his/her own health, and may result in a higher adherence to lifestyle changes resulting in a reduced residual risk. However, robust evidence has yet to be gathered in prospective clinical trials. In addition, wearable technology will greatly enhance the amount of data collected from large populations, enabling the use of big data and AI in precision medicine. Advantages of big data and AI are the opportunity to examine properties of specific groups, such as minorities, without the presence of systematic biases, leading to fair algorithms (212). The current clinical practice is compartmentalized, with healthcare professionals excelling in their respective areas of specialization. Nonetheless, enhancing patient outcomes requires dismantling this siloed approach of working, allowing healthcare professionals to engage in effective communication with both the patient and each other, making the patient the central focus (Figure 1). Supported by laboratory diagnostic professionals, clinical decision support systems, lifestyle coaching, and AI, we can address residual CVD risk and make significant progress towards improved patient care.

### 4.1. Can residual risk be eliminated?

**Figure 2:**
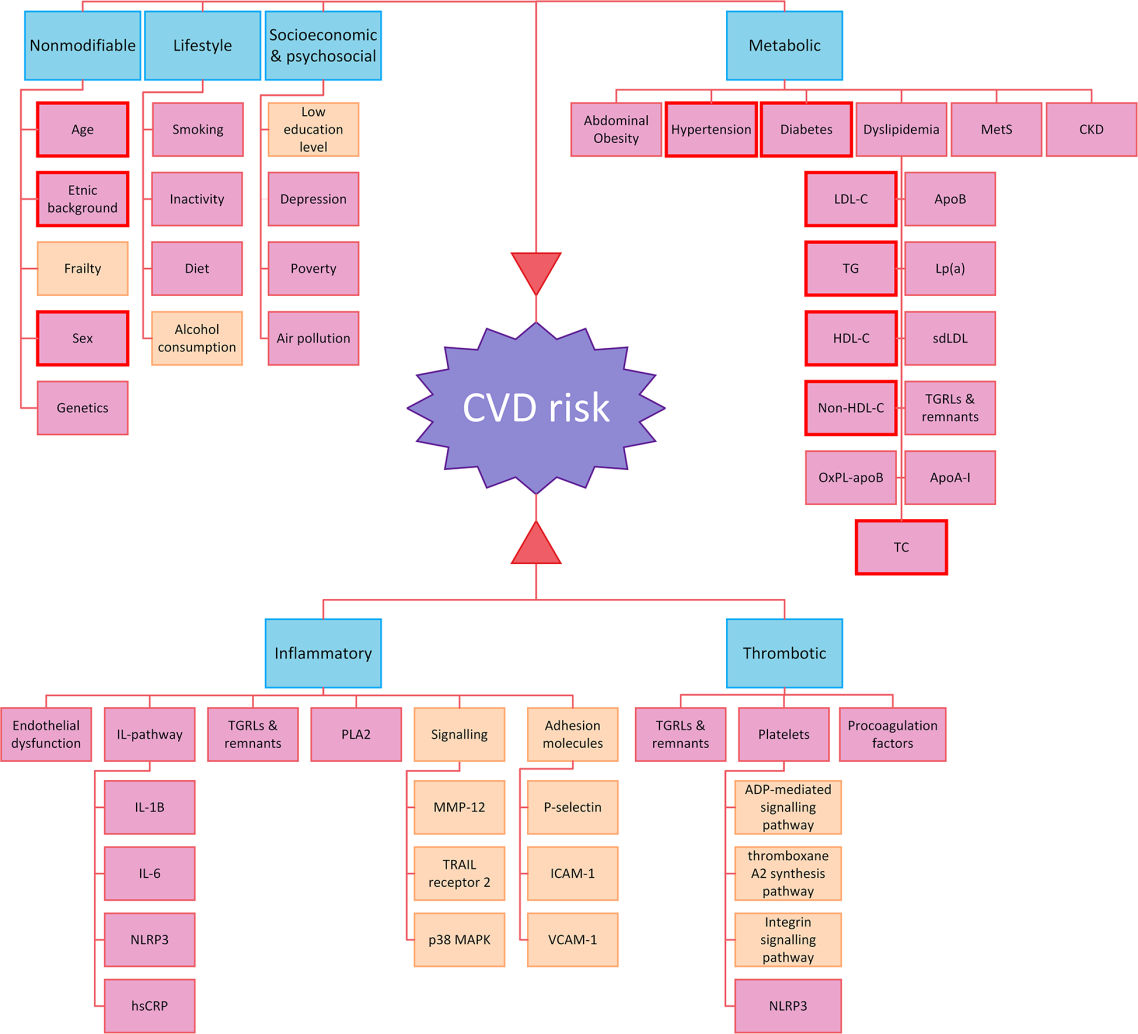
Risk factors contributing to CV risk. Risk factors highlighted in pink were discussed in this review, the ones not discussed in the review but identified through literature search are shown in soft orange. The risk factors considered in clinical risk assessment based on SCORE are depicted with red borders. (219) Apo, apolipoprotein; ADP, adenosine diphosphate; CKD, chronic kidney disease; HDL, high-density lipoprotein; hsCRP, high sensitivity c-reactive protein; ICAM, intercellular adhesion molecules; IL, interleukin; LDL, low-density lipoprotein; MetS, metabolic syndrome; MMP-12, metalloproteinase-12; NLRP3, nucleotide-binding leucine-rich repeat receptor family pyrin domain containing 3; OxPL, oxidized phospholipids; PLA2, phospholipase A2; sdLDL, small dense LDL; TG, triglycerides; TGRL, triglyceride-rich lipoprotein; VCAM-1, vascular cell adhesion protein 1.

While significant progress has been made in reducing the traditional risk factors, emerging risk factors such as Lp(a), inflammation, genetic factors, and psychosocial factors continue to contribute to residual risk. As we gain more knowledge, addressing CV risk becomes increasingly complex, as it involves a multifaceted interplay of various risk factors, including novel risk factors (Figure 2). Even with treatment of all known risk factors, eliminating residual CVD risk is impossible. Shapiro et al. distinguished that only a part of the risk has been treated, the rest being the “*traditional residual risk*” that could be divided into the “*real*” residual risk (that awaits further therapy) and the “*unmodifiable*” risk, which is the risk we cannot eliminate (213).

## 5. Conclusion

Residual CVD risk cannot be eliminated completely. Nevertheless, to diminish residual CVD risk and improve patient management, a paradigm shift from a reductionistic approach towards a holistic approach is necessary. This requires the involvement of laboratory specialists to enable precision diagnostics as a fundament for precision medicine (214). Moving towards P5 medicine for each individual patient, a personalized treatment dependent on their CVD risk and respective lipid profile should be configured. A head-start can be conducted by measuring Lp(a) once in a lifetime (215) and apoB instead of LDL-C in case of aggressive lipid-lowering therapy (214), whereas the measurement of other lipoproteins and apolipoproteins offers the opportunity to molecularly define the (patho)biological profile enabling more precise indications for targeted treatment strategies (216–218). For example, promising results have been demonstrated with ASOs targeted at apoC-III (84, 85). Finally, whereas the empowered patient should take the lead in CVD prevention through lifestyle modification, a patient prone to CVD needs effective medical care and a comprehensive multidisciplinary approach becomes imperative. The latter approach necessitates the collaboration of a diverse range of healthcare professionals, who contribute with their specialized knowledge and expertise to ensure optimal patient care.

## Nomenclature

### Nonstandard Abbreviations and Acronyms

4S: Scandinavian Simvastatin Survival Study
AA: arachidonic acid
ACC: acetyl-CoA carboxylase
ACCORD: Action To Control Cardiovascular Risk In Diabetes
ACS: acute coronary syndrome
ADP: adenosine diphosphate
AF: arterial fibrillation
AFCAPS/TexCAPS: Air Force/Texas Coronary Atherosclerosis Prevention Study
AI: artificial intelligence
AIM-HIGH: Atherothrombosis Intervention In Metabolic Syndrome With Low HDL/High Triglycerides And Impact On Global Health Outcomes
AKI: acute kidney injury
AMI: acute myocardial infarction
ANGPTL: angiopoietin-like protein
Apo: apolipoprotein
APPROACH: A Study Of Volanesorsen (Formerly IONIS-ApoCIIIrx) In Patients With Familial Chylomicronemia Syndrome
ARBITER-2: Arterial Biology For The Investigation Of The Treatment Effects Of Reducing Cholesterol
ASCVD: atherosclerotic cardiovascular disease
ASO: antisense oligonucleotide
ATP: adenosine triphosphate
BMI: body mass index
BNP: brain natriuretic peptide
CABG: coronary artery bypass graft
CAD: coronary artery disease
CANTOS: Canakinumab Anti-Inflammatory Thrombosis Outcomes Study
CARAT: CER-001 Atherosclerosis Regression Acute Coronary Syndrome Trial
CCTA: coronary computed tomography angiography
CETP: cholesterol ester transfer protein
CHD: coronary heart disease
CHIP: clonal hematopoiesis of indeterminate potential
CIRT: Cardiovascular Inflammation Reduction Trial
CKD: chronic kidney disease
CM: chylomicron
COLCOT: Colchicine Cardiovascular Outcomes Trial
COMPASS: Rivaroxaban For The Prevention Of Major Cardiovascular Events In Coronary Or Peripheral Artery Disease
CRP: C-reactive protein
CV: cardiovascular
CVD: cardiovascular disease
DAPT: dual antiplatelet therapy
DART: diet and reinfarction trial
DHA: docosahexaenoic acid
DPI: dual pathway inhibition
EAS: European Atherosclerosis Society
EMA: European Medicine Agency
eNOS: endothelial nitric oxide synthase
ESC: European Society of Cardiology
EURIKA: European Study Of Cardiovascular Risk
FA: fatty acid
FAI: fat attenuation index
FCS: familial chylomicronemia syndrome
FDA: Food & Drug Administration
FH: familial hypercholesterolemia
FIELD: Fenofibrate Intervention And Event Lowering In Diabetes
FMD-J: Flow-Mediated Dilatation—Japan
FOURIER: Further Cardiovascular Outcomes Research With PCSK9 Inhibition In Subjects With Elevated Risk
GalNAc: N-acetylgalactosamine
GLP-1 RA: glucagon-like peptide-1 receptor agonist
GPIHBP1: glycosylphosphatidylinositol-anchored high-density lipoprotein-binding protein-1
HbA1c: hemoglobin A1c
HDL: high-density lipoprotein
HL: hepatic lipase
HMG-CoA: hydroxymethylglutaryl CoA (HMG-CoA)
HRP: high-risk plaque
hsCRP: high sensitivity C-reactive protein
HTG: hypertriglyceridemia
ICAM: intercellular adhesion molecules
IDL: intermediate-density lipoprotein
IHD: ischemic heart disease
IL: interleukin
ILLUMINATE: A Study Examining Torcetrapib/Atorvastatin And Atorvastatin Effects On Clinical CV Events In Patients With Heart Disease
IMPROVE-IT: Improved Reduction Of Outcomes: Vytorin Efficacy International Trial
IMT: Intima-media thickness
iNOS: inducible nitic oxide synthase
INTERHEART: Effect Of Potentially Modifiable Risk Factors Associated With Myocardial Infarction In 52 Countries
IVUS: intravascular ultrasound
JUPITER: Justification For The Use Of Statins In Prevention: An Intervention Trial Evaluating Rosuvastatin
LA: lipoprotein apheresis
LDL: low-density lipoprotein
LMF1: lipase maturation factor-1
LoDoCo: Low-Dose Colchicine
Lp(a): lipoprotein(a)
Lp(a)HORIZON: Assessing The Impact Of Lipoprotein (a) Lowering With Pelacarsen (TQJ230) On Major Cardiovascular Events In Patients With Cardiovascular Disease
LPL: lipoprotein lipase
LRP1: LDL receptor-related protein 1
LXR: liver X receptor
mAb: monoclonal antibody
MACCE: major adverse cardiac and cerebrovascular events
MACE: major adverse cardiac events
MetS: metabolic syndrome
MI: myocardial infarction
MINOCA: myocardial infarction with non-obstructive coronary arteries
MMP-12: metalloproteinase-12
MTP: mitochondrial triglyceride transfer protein
NADPH: nicotinamide adenine dinucleotide phosphate (reduced)
NCP1L1: Niemann-Pieck C1-like1 protein
NF*κβ*: nuclear factor kappa-light-chain-enhancer of activated B cells
NHANES: National Health And Nutrition Examination Survey
NLRP3: nucleotide-binding leucine-rich repeat receptor family pyrin domain containing 3
NMR: nuclear magnetic resonance
NO: nitric oxide
OCEAN(a): Olpasiran Trials of Cardiovascular Events and Lipoprotein(a) Reduction
OxPL: oxidized phospholipids
P5: predictive, preventive, personalized, participatory, psychocognitive
PAR: population attributable risk
PCI: percutaneous coronary intervention
PCOS: polycystic ovary syndrome
PCSK9: proprotein convertase subtilisin/kexin type 9
PEGASUS-TIMI 54: Prevention Of Cardiovascular Events In Patients With Prior Heart Attack Using Ticagrelor Compared To Placebo On A Background Of Aspirin–Thrombolysis In Myocardial Infarction 54
PLA2: phospholipase A2
PLG: plasminogen
PM: particulate matter
PPAR: peroxisome proliferator-activated receptors
PPC: polyene phosphatidylcholine
PREDIMED: Primary Prevention Of Cardiovascular Disease With A Mediterranean Diet
PRIME: Prospective Epidemiological Study of Myocardial Infarction
PROMINENT: Pemafibrate To Reduce Cardiovascular Outcomes By Reducing Triglycerides In Patients With Diabetes
PROVE-IT: Pravastatin Or Atorvastatin Evaluation And Infection Therapy
PROVE-IT-TIMI 22: The Pravastatin Or Atorvastatin Evaluation And Infection Therapy—Thrombolysis In Myocardial Infarction 22
R3I: Residual Risk Reduction Initiative
RAS: renin aldosterone system
REDUCE-IT: Reduction Of Cardiovascular Events With Icosapent Ethyl–Intervention Trial
REVERSAL: Reversal Of Atherosclerosis With Aggressive Lipid Lowering
RNS: reactive nitrogen species
ROS: reactive oxygen species
SATURN: Statins Use In Intracerebral Hemorrhage Patient
sdLDL: small dense LDL
SGLT2i: sodium/glucose cotransporter 2 inhibitor
siRNA: small interfering RNA
SNP: single nucleotide polymorphism
SOLID-TIMI 52: The Stabilization Of Plaques Using Darapladib-Thrombolysis In Myocardial Infarction 52
SPIRE1/SPIRE2: The Evaluation Of Bococizumab (Pf-04950615;Rn316) In Reducing The Occurrence Of Major Cardiovascular Events In High Risk Subjects
sPLA: secretory phospholipase A2
STABILITY: Stabilization Of Atherosclerotic Plaque By Initiation Of Darapladib Therapy
T1DM: type 1 diabetes mellitus
T2DM: type 2 diabetes mellitus
TC: total cholesterol
TG: triglycerides
TGRL: triglyceride-rich lipoprotein
TIMI-22: Pravastatin or Atorvastatin Evaluation and Infection Therapy
TMAO: trimethylamine N-oxide
VCAM-1: vascular cell adhesion protein 1
VISTA-16: Varespladib And Cardiovascular Events In Patients With An Acute Coronary Syndrome 16
VLDL: very low-density lipoprotein
VTE: venous thromboembolism
WC: waist circumference
WHR: waist-to-hip ratio
ZEUS: A Research Study to Look at How Ziltivekimab Works Compared to Placebo in People With Cardiovascular Disease, Chronic Kidney Disease and Inflammation
