# Supplementary material for: High residual cardiovascular risk after lipid-lowering: prime time for Predictive, Preventive, Personalized, Participatory, and Psycho-cognitive medicine
Source: Front Cardiovasc Med. 2023 Oct 16;10:1264319. doi: 10.3389/fcvm.2023.1264319 (PMC10613690; doi:10.3389/fcvm.2023.1264319)
Supplement: Supplementary file 1 [file Table1.docx]

Supplementary Material

# Supplementary Figures and Tables

**Table 1: Risk factors contributing to CV risk and their causality, including the treatment effect on the particular risk factor and the treatment effect on MACE**.

| Risk factor / marker | Causal for CVD? | Treatment | Treatment mechanism | Treatment effect on risk factor/ marker | | Treatment effect on MACE | | Ref |
| --- | --- | --- | --- | --- | --- | --- | --- | --- |
|  |  |  |  | Study population | Effect size | Study population | Effect size |  |
| LDL-c | Yes | Statins | HMG-CoA reductase inhibitor | Hypercholesterolemic, mean baseline LDL-C 3.39-4.91 mmol/L | -50% | Hypercholesterolemic, mean baseline LDL-C 3.39-4.91 mmol/L | -20-35% | ^1-9^ |
|  |  | Alirocumab | mAb against PCSK9 | ACS, baseline LDL-C ≥1.80 mmol/L, on lipid-lowering therapy | -62-66% | ACS, baseline LDL-C ≥1.80 mmol/L, on lipid-lowering therapy | -1.6% (ARR) | ^2, 10^ |
|  |  | Ezetimibe | cholesterol-absorption inhibitor | Hypercholesterolemic, mean baseline LDL-C 1.29-3.17 mmol/L, statin-treated | -20-25% | Hypercholesterolemic, mean baseline LDL-C 1.29-3.17 mmol/L, statin-treated | -7-13% | ^11, 12^ |
|  |  | Inclisiran | siRNA targeting PCSK9 | ASCVD or at high CV risk with elevated LDL-C serum levels, mean baseline LDL-C 2.68-3.92 mmol/L, on lipid-lowering therapy | -51% | Hypercholesterolemic, mean baseline LDL-C 2.68-3.92 mmol/L, on lipid-lowering therapy | -24% | ^12, 13^ |
|  |  | Bempedoic acid | ACC and ATP-citrate lyase inhibitor | ASCVD and/or heFH, mean baseline LDL-C 2.68 mmol/L in statin-treated Hypercholesterolemic, mean baseline LDL-C 3.59 mmol/L, ezetimibe-treated and statin-intolerant | -17%   -29% | Hypercholesterolemic, mean baseline LDL-C 3.59 mmol/L, ezetimibe-treated and statin-intolerant | -13-23% | ^11, 12, 14-17^ |
|  |  | Mipomersen | ASO directed at apoB-100 | Mild dyslipidemic, mean baseline LDL-C 3.31 mmol/L heFH, mean baseline LDL-C 4.50 mmol/L, on lipid-lowering therapy | -35%  -21-34% | - | Unknown | ^2, 18, 19^ |
|  |  | Lomitapide | MTP inhibitor | hoFH mean baseline LDL-C 8.69 mmol/L, on lipid-lowering therapy | -38-50% | - | Unknown | ^2, 20^ |
|  |  | Evinacumab | mAb against ANGPTL3 | HTG, mean baseline LDL-C 3.17-3.75 mmol/L hoFH, mean baseline LDL-C 8.32 mmol/L, on lipid-lowering therapy | -23%  -47% | - | Unknown | ^21-23^ |
| ApoB | Potentially (CHD) | Mipomersen | ASO directed at apoB-100 | Mild dyslipidemic, mean baseline LDL-C 2.61-2.92 mmol/L Severe-hypercholesterolemic, mean baseline apoB 1.8 mmol/L, on lipid-lowering therapy heFH, mean baseline ApoB 1.52 g/L, on lipid-lowering therapy | -50%  -36%   -23-33% | - | Unknown | ^2, 18, 19, 24, 25^ |
|  |  | Lomitapide | MTP inhibitor | hoFH, mean baseline ApoB 2.6 g/L, on lipid-lowering therapy | -49% | - | Unknown | ^20, 26^ |
|  |  | Alirocumab | mAb against PCSK9 | ACS, baseline ApoB <0.75, 0.75–<0.90, ≥0.90 g/L, on lipid-lowering therapy | -42-54% | ACS, baseline ApoB <0.75, 0.75–<0.90, ≥0.90 g/L, on lipid-lowering therapy | -1.6%  (ARR) | ^10^ |
|  |  | Bempedoic acid | ACC and ATP-citrate lyase inhibitor | ASCVD and/or heFH, mean baseline ApoB 0.87 g/L, statin-treated | -13% | Statin-intolerant | -13-23% | ^14, 15^ |
|  |  | Fibrates | PPAR-α activator | Hypercholesterolemic | -10% | With and without known history of CVD | -22% | ^12, 27, 28^ |
|  |  | Saroglitazar | PPARα/γ agonist | Diabetic dyslipidemic, mean baseline ApoB 1.01 g/L, statin-treated | -32% | - | Unknown | ^29^ |
|  |  | Evinacumab | mAb against ANGPTL3 | hoFH, on lipid-lowering therapy | -46% | - | Unknown | ^21^ |
| Lp(a) | Yes (CVD, aortic valve stenosis) | Lipoprotein apheresis | intervention | Mean baseline LDL-C of 2.07 mmol/L and Lp(a) of 138 mg/dL on lipid-lowering therapy | -63% | Lp(a) > 60 mg/dL, normal LDL-C, and CVD | >70% | ^30-33^ |
|  |  | Alirocumab | mAb against PCSK9 | ACS, baseline LDL-C ≥1.80 mmol/L, on lipid-lowering therapy | -23% | ACS, baseline LDL-C ≥1.80 mmol/L, on lipid-lowering therapy | -1.6% (ARR) | ^10, 34^ |
|  |  | Pelacarsen | ASO directed at apo(a) | CVD, on lipid-lowering therapy Healthy | -35-80% -90% | CVD, on lipid-lowering therapy | Results expected in 2025 | ^35-37^ |
|  |  | Mipomersen | ASO directed at apoB-100 | heFH and CHD, on lipid-lowering therapy FH, on lipid lowering therapy | -21%  -31% | - | Unknown | ^34, 38^ |
|  |  | Lomitapide | MTP inhibitor | hoFH, on lipid-lowering therapy | -13% | - | Unknown | ^26, 34^ |
|  |  | Evolocumab | mAb against PCSK9 | ASCVD, on lipid-lowering therapy | -27% | ASCVD, on lipid-lowering therapy | -16% | ^11, 39^ |
|  |  | Olpasiran | siRNA against apo(a) | ASCVD, on lipid-lowering therapy | -71-100% | ASCVD and elevated Lp(a) | Results expected in 2027 | ^40^ |
| HDL-C | No  (CHD, MI, IHD) | Aerobic exercise | lifestyle intervention | Sedentary lifestyle without CVD | +5-10% | General population | ≤-50% | ^41-44^ |
|  |  | Smoking cessation | lifestyle intervention | Cigarette smokers | +0.1 mmol/L | Acute ischemic stroke | -14% | ^44, 45^ |
|  |  | Apabetalone  (RVX-208) | apoA-I mimetics | Stable CAD, statin-treated Symptomatic CAD with low HDL-C levels, statin-treated | +3-8% No effect | - | Unknown | ^26^ |
| ApoA-I | Likely not (CAD) | CSL112 | apoA-I mimetics | MI, statin-treated | +123% | - | Unknown | ^46, 47^ |
|  |  | Apabetalone  (RVX-208) | apoA-I mimetics | Stable CAD, statin-treated Symptomatic CAD with low HDL-C levels, statin-treated | +5.6-10% No effect | - | Unknown | ^26, 48^ |
| TG | Yes  (CHD) | Nicotinic acid  (niacin) | Reduces hepatic TG synthesis | CHD with HDL-C <1.16 mmol/L, statin-treated | -13% | HDL-C <1.81 mmol/L or at high CV risk, statin-treated | No effect | ^49-51^ |
|  |  | Fibrates | PPAR-α activator, inhibits ApoC-III expression, inhibits VLDL synthesis and secretion, stimulates LPL expression | IIB phenotype of Fredrickson classification Hypercholesterolemic | -30-50%  ≤-30% | CAD | -24% | ^27, 28^ |
|  |  | Fenofibrate | PPAR-α activator, reduces ApoC-III, LPL activator | T2DM, statin-treated | -22% | T2DM, statin-treated  TG≥ 2.31 mmol/L and HDL-C≤0.88 mmol/L, statin-treated | No effect -31% | ^27^ |
|  |  | Pemafibrate (K-877) | SPPARMα modulator | Dyslipidemic T2DM, mild-to-moderate HTG, and HDL-C <1.03 mmol/L, on lipid-lowering therapy and statin-intolerant | -42.7% -26.2% | T2DM, mild-to-moderate HTG, and HDL-C <1.03 mmol/L, on lipid-lowering therapy and statin-intolerant | No effect | ^12, 52^ |
|  |  | Saroglitazar | PPARα/γ agonist | Diabetic dyslipidemic, statin-treated | -45% | - | Unknown | ^29^ |
|  |  | EPA | ω3-fatty acid | Hypercholesterolemic, statin-treated | -9% | Hypercholesterolemic, statin-treated | -19% | ^53-55^ |
|  |  | EPA+DHA | ω3-fatty acid | MI, preventive treatments HTG, statin-treated | -3% -19% | MI, preventive treatments DM without CVD HTG, statin-treated | -30%  No effect No effect | ^55, 56^ |
|  |  | Icosapent ethyl (IPE) | stable ethyl ester of EPA | Hypercholesterolemic, statin-treated High CV risk and elevated TG, statin-treated | -9% -17% | HTG, statin-treated | -25% | ^56^ |
|  |  | Volanesorsen (ISIS-APOCIIIRx) | ASO directed at ApoC-III | FCS | -77% | - | Unknown | ^21, 46, 57^ |
|  |  | Olezarsen (AKCEA-APOCIII-LRx) | ASO directed at ApoC-III | Healthy, mildly elevated TG | -77% | - | Unknown | ^57^ |
|  |  | Evinacumab | mAb against ANGPTL3 | HTG hoFH, on lipid-lowering therapy | -76.9% -47% | - | Unknown | ^21^ |
|  |  | AKCEA-ANGPTL3-LRx | ASO directed at ANGPTL3 | Healthy | -49% | - | Unknown | ^21, 26^ |
| TGRLs | Yes  (CHD and aortic valve stenosis) | Mipomersen | ASO directed at apoB-100 | hoFH, on lipid-lowering therapy | -17% VLDL-C | - | Unknown | ^38, 58, 59^ |
|  |  | Olezarsen | ASO directed at ApoC-III | Moderate HTG and at high CV risk, on lipid-lowering therapy | -58% VLDL-C | - | Unknown | ^60^ |
|  |  | Saroglitazar | PPARα/γ agonist | Diabetic dyslipidemic, statin-treated | -45% VLDL-C | - | Unknown | ^29^ |
|  |  | Pemafibrate (K-877) | SPPAR-α modulator | T2DM, mild-to-moderate HTG, and HDL-C <1.03 mmol/L, on lipid-lowering therapy and statin-intolerant | -26% VLDL-C | T2DM, mild-to-moderate HTG, and low HDL-C and LDL-C levels | No effect | ^52^ |
|  |  | Volanesorsen | ASO directed at ApoC-III | FCS | -58% VLDL-C | - | Unknown | ^21^ |
| Remnant-C | Yes  (IHD, aortic valve stenosis) | Pemafibrate (K-877) | SPPAR-α modulator | T2DM, mild-to-moderate HTG, and HDL-C <1.03 mmol/L, on lipid-lowering therapy and statin-intolerant | -26% | T2DM, mild-to-moderate HTG, and low HDL-C and LDL-C levels | No effect | ^52, 58, 61, 62^ |
| ApoC-III | Yes  (CHD) | Volanesorsen | ASO directed at apoC-III | FCS | -84% | - | Unknown | ^46, 57, 59, 63^ |
|  |  | Olezarsen | ASO directed at apoC-III | Mildly elevated TG  Moderate HTG and at high CV risk lipid-lowering therapy | -92%  -74% | - | Unknown | ^57, 60^ |
|  |  | Pemafibrate (K-877) | SPPAR-α modulator | T2DM, mild-to-moderate HTG, and HDL-C <1.03 mmol/L, on lipid-lowering therapy and statin-intolerant | -28% | T2DM, mild-to-moderate HTG, and HDL-C <1.03 mmol/L, on lipid-lowering therapy and statin-intolerant | No effect | ^52^ |
| ANGPTL3 | Yes  (CVD) | Evinacumab | mAb against ANGPTL3 | - | Unknown | - | Unknown | ^21, 64, 65^ |
|  |  | AKCEA-ANGPTL3-LRx | ASO directed at ANGPTL3 | Healthy | -47-82% | - | Unknown | ^21, 26, 64^ |
|  |  | ARO-ANG3 | siRNA targeting ANGPTL3 | Healthy | -55-83% | - | Unknown | ^21, 64^ |
| OxPL | Unknown | Pelacarsen | ASO directed at apo(a) | CVD, on lipid-lowering therapy | -30-90% |  | Results expected in 2025 | ^36, 66^ |
| PLA2 | No  (CHD) | Darapladib | Lp-PLA2i | CHD, statin-treated | -43-66% | ACS, on lipid-lowering therapy Stable CHD, on lipid-lowering therapy | No effect No effect | ^67-70^ |
|  |  | Varespladib | sPLA2i | - | Unknown | ACS <96 h prior | Increased risk | ^71^ |
| IL-6 | Indirectly, via IL-6 receptor for CHD, AF, and stroke | Darapladib | Lp-PLA2i | ACS, on lipid-lowering therapy | -12% | ACS, on lipid-lowering therapy | No effect | ^71-75^ |
|  |  | Methotrexate | folic acid antagonist | ASCVD | No effect | ASCVD | No effect | ^76, 77^ |
|  |  | Bempedoic acid | ACC and ATP-citrate lyase inhibitor | ASCVD and/or heFH, statin-treated | -4% | statin-intolerant | -13-23% | ^14, 15^ |
|  |  | Canakinumab | mAb against IL-1β receptor | Stable post-MI hsCRP levels>2mg/L, on lipid-lowering therapy | -20-40% | on-treatment hsCRP<2mg/L | -26% | ^78^ |
| IL-1 | Indirectly as part of IL-6 pathway | Methotrexate | folic acid antagonist | ASCVD | No effect | ASCVD | No effect | ^76, 77^ |
| hsCRP | No | Darapladib | Lp-PLA2i | CHD, statin-treated ACS, on lipid-lowering therapy | -20% -13% | ACS, on lipid-lowering therapy Stable CHD, on lipid-lowering therapy | No effect No effect | ^71, 75^ |
|  |  | Varespladib | sPLA2i | Stable CHD, statin-treated | -55.6% | ACS <96 h prior | Increased risk | ^71^ |
|  |  | Methotrexate | folic acid antagonist | ASCVD | No effect | ASCVD | No effect | ^76, 77^ |
|  |  | Bempedoic acid | ACC and ATP-citrate lyase inhibitor | ASCVD and/or heFH, statin-treated | -27% | Statin-intolerant | -13-23% | ^14, 15^ |
|  |  | Colchicine | inhibits NLRP3 inflammasome activation | ACS, statin-treated | -37.3% | ACS, statin-treated Chronic coronary disease | -23%  -30% | ^79-81^ |
|  |  | Canakinumab | mAb against IL-1β receptor | Stable post-MI hsCRP levels>2mg/L, on lipid-lowering therapy  T2DM | -20-40%  -36-65% | Stable post-MI on-treatment hsCRP<2mg/L, on lipid-lowering therapy | -26% | ^71, 78^ |
|  |  | Ziltivekimab | mAb against IL-6 | Elevated hsCRP and CKD | -77-92% | Unknown | - | ^82^ |
| Platelet-related |  | Rivaroxaban | Factor Xa inhibitor | - | - | ACS, in addition to standard therapy ASCVD, aspirin-treated Post-MI with stable CAD, aspirin-treated | -16% -24% -22% | ^83, 84^ |
|  |  | Clopidogrel | nhibits the binding of ADP | - | - | ASCVD Prior MI, ischemic stroke, or symptomatic PAD, aspirin-treated | -8.7% -17% | ^85, 86^ |
|  |  | Ticagrelor | P2Y12 inhibitor | - | - | ACS with stable CAD, aspirin-treated | -16% | ^84^ |
| Obesity | Controversial | Bariatric surgery | intervention | Adolescents | -30% BMI | Patients that underwent bariatric surgery | -27-42.1% | ^43, 87^ |
|  |  | Mediterranean diet | intervention | At high CV risk, without pre-existing CVD | Yes | Compared to low-fat diet in population at high CV risk, without pre-existing CVD | -30% | ^88, 89^ |
| Poor diet | Yes | Mediterranean diet | lifestyle intervention | At high CV risk, without pre-existing CVD | Yes | Compared to low-fat diet in population at high CV risk, without pre-existing CVD | -30% | ^88, 89^ |
| Inactivity | Controversial | Aerobic exercise | lifestyle intervention | Yes | Yes | General population | ≤-50% | ^43^ |
| Smoking | Yes (CVD) | Smoking cessation | lifestyle intervention | Yes | Yes | Acute ischemic stroke | -14% | ^44, 45^ |

ACS; acute coronary syndrome, AF; arterial fibrillation, ANGPTL; angiopoietin-like protein, Apo; apolipoprotein, mAb; monoclonal antibody, ASCVD; atherosclerotic cardiovascular disease, ASO; antisense oligonucleotide, ATP; adenosine triphosphate, CAD; coronary artery disease, CHD; coronary heart disease, CKD; chronic kidney disease, CVD; cardiovascular disease, FCS; familial chylomicronemia syndrome, FH; familial hypercholesterolemia, HDL; high-density lipoprotein, hsCRP; high sensitivity c-reactive protein, HTG; hypertriglyceridemia, IL; interleukin, LDL; low-density lipoprotein, Lp(a); lipoprotein (a), MI; myocardial infarction, MTP; mitochondrial triglyceride transfer protein, OxPL; oxidized phospholipids, siRNA; small interfering RNA, ACC; acetyl-CoA carboxylase, PCSK9; proprotein convertase subtilisin/kexin type 9, PLA2; phospholipase A2, PPAR; peroxisome proliferator-activated receptors, sPLA; secretory phospholipase A2, T2DM; type 2 diabetes mellitus, TG; triglycerides, TGRL; triglyceride-rich lipoprotein, VLDL; very low-density lipoprotein.

1. Libby P. The forgotten majority: unfinished business in cardiovascular risk reduction. *J Am Coll Cardiol*. 2005;46:1225-8.

2. Ahn CH and Choi SH. New drugs for treating dyslipidemia: beyond statins. *Diabetes Metab J*. 2015;39:87-94.

3. Vanuzzo D. The epidemiological concept of residual risk. *Intern Emerg Med*. 2011;6 Suppl 1:45-51.

4. Randomised trial of cholesterol lowering in 4444 patients with coronary heart disease: the Scandinavian Simvastatin Survival Study (4S). *Lancet*. 1994;344:1383-9.

5. Group LS. Long-term effectiveness and safety of pravastatin in 9014 patients with coronary heart disease and average cholesterol concentrations: the LIPID trial follow-up. *Lancet*. 2002;359:1379-87.

6. Sacks FM, Pfeffer MA, Moye LA, Rouleau JL, Rutherford JD, Cole TG, Brown L, Warnica JW, Arnold JMO, Wun C-C, Davis BR and Braunwald E. The Effect of Pravastatin on Coronary Events after Myocardial Infarction in Patients with Average Cholesterol Levels. *N Engl J Med*. 1996;335:1001-1009.

7. Heart Protection Study Collaborative G. MRC/BHF Heart Protection Study of cholesterol lowering with simvastatin in 20,536 high-risk individuals: a randomised placebo-controlled trial. *Lancet*. 2002;360:7-22.

8. Shepherd J, Cobbe SM, Ford I, Isles CG, Lorimer AR, Macfarlane PW, McKillop JH and Packard CJ. Prevention of Coronary Heart Disease with Pravastatin in Men with Hypercholesterolemia. *N Engl J Med*. 1995;333:1301-1308.

9. Downs JR, Clearfield M, Weis S, Whitney E, Shapiro DR, Beere PA, Langendorfer A, Stein EA, Kruyer W, Gotto JAM and for the ATRG. Primary Prevention of Acute Coronary Events With Lovastatin in Men and Women With Average Cholesterol LevelsResults of AFCAPS/TexCAPS. *JAMA*. 1998;279:1615-1622.

10. Hagström E, Steg PG, Szarek M, Bhatt DL, Bittner VA, Danchin N, Diaz R, Goodman SG, Harrington RA, Jukema JW, Liberopoulos E, Marx N, McGinniss J, Manvelian G, Pordy R, Scemama M, White HD, Zeiher AM and Schwartz GG. Apolipoprotein B, Residual Cardiovascular Risk After Acute Coronary Syndrome, and Effects of Alirocumab. *Circulation*. 2022;146:657-672.

11. Berman AN and Blankstein R. Optimizing Dyslipidemia Management for the Prevention of Cardiovascular Disease: a Focus on Risk Assessment and Therapeutic Options. *Curr Cardiol Rep*. 2019;21:110.

12. Bove M, Cicero AFG and Borghi C. Emerging drugs for the treatment of hypercholesterolemia. *Expert Opin Emerg Drugs*. 2019;24:63-69.

13. Khan SA, Naz A, Qamar Masood M and Shah R. Meta-Analysis of Inclisiran for the Treatment of Hypercholesterolemia. *Am J Cardiol*. 2020;134:69-73.

14. Ridker PM, Lei L, Ray KK, Ballantyne CM, Bradwin G and Rifai N. Effects of bempedoic acid on CRP, IL-6, fibrinogen and lipoprotein(a) in patients with residual inflammatory risk: A secondary analysis of the CLEAR harmony trial. *J Clin Lipidol*. 2023;17:297-302.

15. Nissen SE, Lincoff AM, Brennan D, Ray KK, Mason D, Kastelein JJP, Thompson PD, Libby P, Cho L, Plutzky J, Bays HE, Moriarty PM, Menon V, Grobbee DE, Louie MJ, Chen C-F, Li N, Bloedon L, Robinson P, Horner M, Sasiela WJ, McCluskey J, Davey D, Fajardo-Campos P, Petrovic P, Fedacko J, Zmuda W, Lukyanov Y and Nicholls SJ. Bempedoic Acid and Cardiovascular Outcomes in Statin-Intolerant Patients. *N Engl J Med*. 2023;388:1353-1364.

16. Ballantyne CM, Banach M, Mancini GBJ, Lepor NE, Hanselman JC, Zhao X and Leiter LA. Efficacy and safety of bempedoic acid added to ezetimibe in statin-intolerant patients with hypercholesterolemia: A randomized, placebo-controlled study. *Atherosclerosis*. 2018;277:195-203.

17. Ray KK, Bays HE, Catapano AL, Lalwani ND, Bloedon LT, Sterling LR, Robinson PL and Ballantyne CM. Safety and Efficacy of Bempedoic Acid to Reduce LDL Cholesterol. *N Engl J Med*. 2019;380:1022-1032.

18. Kastelein JJP, Wedel MK, Baker BF, Su J, Bradley JD, Yu RZ, Chuang E, Graham MJ and Crooke RM. Potent Reduction of Apolipoprotein B and Low-Density Lipoprotein Cholesterol by Short-Term Administration of an Antisense Inhibitor of Apolipoprotein B. *Circulation*. 2006;114:1729-1735.

19. Akdim F, Visser ME, Tribble DL, Baker BF, Stroes ESG, Yu R, Flaim JD, Su J, Stein EA and Kastelein JJP. Effect of Mipomersen, an Apolipoprotein B Synthesis Inhibitor, on Low-Density Lipoprotein Cholesterol in Patients With Familial Hypercholesterolemia. *Am J Cardiol*. 2010;105:1413-1419.

20. Cuchel M, Meagher EA, du Toit Theron H, Blom DJ, Marais AD, Hegele RA, Averna MR, Sirtori CR, Shah PK, Gaudet D, Stefanutti C, Vigna GB, Du Plessis AME, Propert KJ, Sasiela WJ, Bloedon LT and Rader DJ. Efficacy and safety of a microsomal triglyceride transfer protein inhibitor in patients with homozygous familial hypercholesterolaemia: a single-arm, open-label, phase 3 study. *Lancet*. 2013;381:40-46.

21. Akoumianakis I, Zvintzou E, Kypreos K and Filippatos TD. ANGPTL3 and Apolipoprotein C-III as Novel Lipid-Lowering Targets. *Curr Atheroscler Rep*. 2021;23:20.

22. Ahmad Z, Banerjee P, Hamon S, Chan K-C, Bouzelmat A, Sasiela WJ, Pordy R, Mellis S, Dansky H, Gipe DA and Dunbar RL. Inhibition of Angiopoietin-Like Protein 3 With a Monoclonal Antibody Reduces Triglycerides in Hypertriglyceridemia. *Circulation*. 2019;140:470-486.

23. Gaudet D, Gipe DA, Pordy R, Sasiela W, Chan K-C and Khoury E. Safety and efficacy of evinacumab, a monoclonal antibody to ANGPTL3, in patients with homozygous familial hypercholesterolemia receiving concomitant lipid-lowering therapies. *J Clin Lipidol*. 2016;10:715.

24. Besseling J, Hovingh GK and Stroes ES. Antisense oligonucleotides in the treatment of lipid disorders: pitfalls and promises. *Neth J Med*. 2013;71:118-22.

25. Richardson TG, Sanderson E, Palmer TM, Ala-Korpela M, Ference BA, Davey Smith G and Holmes MV. Evaluating the relationship between circulating lipoprotein lipids and apolipoproteins with risk of coronary heart disease: A multivariable Mendelian randomisation analysis. *PLOS Medicine*. 2020;17:e1003062.

26. Shapiro MD and Fazio S. From Lipids to Inflammation: New Approaches to Reducing Atherosclerotic Risk. *Circ Res*. 2016;118:732-49.

27. Wanner C and Krane V. Recent advances in the treatment of atherogenic dyslipidemia in type 2 diabetes mellitus. *Kidney Blood Press Res*. 2011;34:209-17.

28. Chapman MJ. Fibrates in 2003: therapeutic action in atherogenic dyslipidaemia and future perspectives. *Atherosclerosis*. 2003;171:1-13.

29. Sosale A, Saboo B and Sosale B. Saroglitazar for the treatment of hypertrig-lyceridemia in patients with type 2 diabetes: current evidence. *Diabetes Metab Syndr Obes*. 2015;8:189-96.

30. Clarke R, Peden JF, Hopewell JC, Kyriakou T, Goel A, Heath SC, Parish S, Barlera S, Franzosi MG, Rust S, Bennett D, Silveira A, Malarstig A, Green FR, Lathrop M, Gigante B, Leander K, de Faire U, Seedorf U, Hamsten A, Collins R, Watkins H and Farrall M. Genetic Variants Associated with Lp(a) Lipoprotein Level and Coronary Disease. *N Engl J Med*. 2009;361:2518-2528.

31. Kamstrup PR, Tybjærg-Hansen A, Steffensen R and Nordestgaard BG. Genetically Elevated Lipoprotein(a) and Increased Risk of Myocardial Infarction. *JAMA*. 2009;301:2331-2339.

32. Nordestgaard BG and Langsted A. Lipoprotein (a) as a cause of cardiovascular disease: insights from epidemiology, genetics, and biology. *J Lipid Res*. 2016;57:1953-1975.

33. Moriarty PM, Gray JV and Gorby LK. Lipoprotein apheresis for lipoprotein(a) and cardiovascular disease. *J Clin Lipidol*. 2019;13:894-900.

34. Wu MF, Xu KZ, Guo YG, Yu J, Wu Y and Lin LM. Lipoprotein(a) and Atherosclerotic Cardiovascular Disease: Current Understanding and Future Perspectives. *Cardiovasc Drugs Ther*. 2019;33:739-748.

35. Rhainds D, Brodeur MR and Tardif JC. Lipoprotein (a): When to Measure and How to Treat? *Curr Atheroscler Rep*. 2021;23:51.

36. Tsimikas S. A Test in Context: Lipoprotein(a): Diagnosis, Prognosis, Controversies, and Emerging Therapies. *J Am Coll Cardiol*. 2017;69:692-711.

37. Melita H, Manolis AA, Manolis TA and Manolis AS. Lipoprotein(a) and Cardiovascular Disease: A Missing Link for Premature Atherosclerotic Heart Disease and/or Residual Risk. *J Cardiovasc Pharmacol*. 2022;79:e18-e35.

38. Florentin M, Liberopoulos EN, Mikhailidis DP and Elisaf MS. Emerging options in the treatment of dyslipidemias: a bright future? *Expert Opin Emerg Drugs*. 2011;16:247-70.

39. O'Donoghue ML, Fazio S, Giugliano RP, Stroes ESG, Kanevsky E, Gouni-Berthold I, Im K, Lira Pineda A, Wasserman SM, Češka R, Ezhov MV, Jukema JW, Jensen HK, Tokgözoğlu SL, Mach F, Huber K, Sever PS, Keech AC, Pedersen TR and Sabatine MS. Lipoprotein(a), PCSK9 Inhibition, and Cardiovascular Risk. *Circulation*. 2019;139:1483-1492.

40. O’Donoghue ML, Rosenson RS, Gencer B, López JAG, Lepor NE, Baum SJ, Stout E, Gaudet D, Knusel B, Kuder JF, Ran X, Murphy SA, Wang H, Wu Y, Kassahun H and Sabatine MS. Small Interfering RNA to Reduce Lipoprotein(a) in Cardiovascular Disease. *N Engl J Med*. 2022;387:1855-1864.

41. Park S, Han K, Lee S, Kim Y, Lee Y, Kang MW, Park S, Kim YC, Han SS, Lee H, Lee JP, Joo KW, Lim CS, Kim YS and Kim DK. Cardiovascular or mortality risk of controlled hypertension and importance of physical activity. *Heart*. 2021;107:1472-1479.

42. Judge EP, Phelan D and O'Shea D. Beyond statin therapy: a review of the management of residual risk in diabetes mellitus. *J R Soc Med*. 2010;103:357-62.

43. English WJ, Spann MD, Aher CV and Williams DB. Cardiovascular risk reduction following metabolic and bariatric surgery. *Ann Transl Med*. 2020;8:S12.

44. Hausenloy DJ and Yellon DM. Targeting residual cardiovascular risk: raising high-density lipoprotein cholesterol levels. *Postgrad Med J*. 2008;84:590-8.

45. Gynnild MN, Hageman SHJ, Dorresteijn JAN, Spigset O, Lydersen S, Wethal T, Saltvedt I, Visseren FLJ and Ellekjær H. Risk Stratification in Patients with Ischemic Stroke and Residual Cardiovascular Risk with Current Secondary Prevention. *Clin Epidemiol*. 2021;13:813-823.

46. Lee CK, Liao CW, Meng SW, Wu WK, Chiang JY and Wu MS. Lipids and Lipoproteins in Health and Disease: Focus on Targeting Atherosclerosis. *Biomedicines*. 2021;9:985.

47. Karjalainen MK, Holmes MV, Wang Q, Anufrieva O, Kähönen M, Lehtimäki T, Havulinna AS, Kristiansson K, Salomaa V, Perola M, Viikari JS, Raitakari OT, Järvelin M-R, Ala-Korpela M and Kettunen J. Apolipoprotein A-I concentrations and risk of coronary artery disease: A Mendelian randomization study. *Atherosclerosis*. 2020;299:56-63.

48. Kosmas CE, Sourlas A, Silverio D, Montan PD and Guzman E. Novel lipid-modifying therapies addressing unmet needs in cardiovascular disease. *World J Cardiol*. 2019;11:256-265.

49. Holmes MV, Asselbergs FW, Palmer TM, Drenos F, Lanktree MB, Nelson CP, Dale CE, Padmanabhan S, Finan C, Swerdlow DI, Tragante V, van Iperen EPA, Sivapalaratnam S, Shah S, Elbers CC, Shah T, Engmann J, Giambartolomei C, White J, Zabaneh D, Sofat R, McLachlan S, on behalf of the Uc, Doevendans PA, Balmforth AJ, Hall AS, North KE, Almoguera B, Hoogeveen RC, Cushman M, Fornage M, Patel SR, Redline S, Siscovick DS, Tsai MY, Karczewski KJ, Hofker MH, Verschuren WM, Bots ML, van der Schouw YT, Melander O, Dominiczak AF, Morris R, Ben-Shlomo Y, Price J, Kumari M, Baumert J, Peters A, Thorand B, Koenig W, Gaunt TR, Humphries SE, Clarke R, Watkins H, Farrall M, Wilson JG, Rich SS, de Bakker PIW, Lange LA, Davey Smith G, Reiner AP, Talmud PJ, Kivimäki M, Lawlor DA, Dudbridge F, Samani NJ, Keating BJ, Hingorani AD and Casas JP. Mendelian randomization of blood lipids for coronary heart disease. *Eur Heart J*. 2015;36:539-550.

50. Taylor AJ, Sullenberger LE, Lee HJ, Lee JK and Grace KA. Arterial Biology for the Investigation of the Treatment Effects of Reducing Cholesterol (ARBITER) 2: a double-blind, placebo-controlled study of extended-release niacin on atherosclerosis progression in secondary prevention patients treated with statins. *Circulation*. 2004;110:3512-7.

51. Jeyamalar R, Wan Azman WA, Nawawi H, Choo GH, Ng WK, Rosli MA, Al Fazir O, Sazzli K, Oteh M and Quek DKL. Updates in the management of Dyslipidaemia in the high and very high risk individual for CV risk reduction. *Med J Malaysia*. 2018;73:154-162.

52. Das Pradhan A, Glynn RJ, Fruchart J-C, MacFadyen JG, Zaharris ES, Everett BM, Campbell SE, Oshima R, Amarenco P, Blom DJ, Brinton EA, Eckel RH, Elam MB, Felicio JS, Ginsberg HN, Goudev A, Ishibashi S, Joseph J, Kodama T, Koenig W, Leiter LA, Lorenzatti AJ, Mankovsky B, Marx N, Nordestgaard BG, Páll D, Ray KK, Santos RD, Soran H, Susekov A, Tendera M, Yokote K, Paynter NP, Buring JE, Libby P and Ridker PM. Triglyceride Lowering with Pemafibrate to Reduce Cardiovascular Risk. *N Engl J Med*. 2022;387:1923-1934.

53. Borow KM, Nelson JR and Mason RP. Biologic plausibility, cellular effects, and molecular mechanisms of eicosapentaenoic acid (EPA) in atherosclerosis. *Atherosclerosis*. 2015;242:357-66.

54. Sperling LS and Nelson JR. History and future of omega-3 fatty acids in cardiovascular disease. *Curr Med Res Opin*. 2016;32:301-11.

55. Perez-Martinez P, Katsiki N and Mikhailidis DP. The Role of n-3 Fatty Acids in Cardiovascular Disease: Back to the Future. *Angiology*. 2020;71:10-16.

56. Mason RP, Sherratt SCR and Eckel RH. Rationale for different formulations of omega-3 fatty acids leading to differences in residual cardiovascular risk reduction. *Metabolism*. 2022;130:155161.

57. Katzmann JL, Packard CJ, Chapman MJ, Katzmann I and Laufs U. Targeting RNA With Antisense Oligonucleotides and Small Interfering RNA: JACC State-of-the-Art Review. *J Am Coll Cardiol*. 2020;76:563-579.

58. Kaltoft M, Langsted A and Nordestgaard BG. Triglycerides and remnant cholesterol associated with risk of aortic valve stenosis: Mendelian randomization in the Copenhagen General Population Study. *Eur Heart J*. 2020;41:2288-2299.

59. Musunuru K and Kathiresan S. Surprises From Genetic Analyses of Lipid Risk Factors for Atherosclerosis. *Circ Res*. 2016;118:579-585.

60. Tardif JC, Karwatowska-Prokopczuk E, Amour ES, Ballantyne CM, Shapiro MD, Moriarty PM, Baum SJ, Hurh E, Bartlett VJ, Kingsbury J, Figueroa AL, Alexander VJ, Tami J, Witztum JL, Geary RS, O'Dea LSL, Tsimikas S and Gaudet D. Apolipoprotein C-III reduction in subjects with moderate hypertriglyceridaemia and at high cardiovascular risk. *Eur Heart J*. 2022;43:1401-1412.

61. Varbo A, Benn M, Tybjærg-Hansen A, Jørgensen Anders B, Frikke-Schmidt R and Nordestgaard Børge G. Remnant Cholesterol as a Causal Risk Factor for Ischemic Heart Disease. *J Am Coll Cardiol*. 2013;61:427-436.

62. Varbo A, Benn M, Tybjærg-Hansen A and Nordestgaard BG. Elevated remnant cholesterol causes both low-grade inflammation and ischemic heart disease, whereas elevated low-density lipoprotein cholesterol causes ischemic heart disease without inflammation. *Circulation*. 2013;128:1298-309.

63. Katzmann JL, Werner CM, Stojakovic T, März W, Scharnagl H and Laufs U. Apolipoprotein CIII predicts cardiovascular events in patients with coronary artery disease: a prospective observational study. *Lipids Health Dis*. 2020;19:116.

64. Hussain A and Ballantyne CM. New Approaches for the Prevention and Treatment of Cardiovascular Disease: Focus on Lipoproteins and Inflammation. *Annu Rev Med*. 2021;72:431-446.

65. Dewey FE, Gusarova V, Dunbar RL, O’Dushlaine C, Schurmann C, Gottesman O, McCarthy S, Van Hout CV, Bruse S, Dansky HM, Leader JB, Murray MF, Ritchie MD, Kirchner HL, Habegger L, Lopez A, Penn J, Zhao A, Shao W, Stahl N, Murphy AJ, Hamon S, Bouzelmat A, Zhang R, Shumel B, Pordy R, Gipe D, Herman GA, Sheu WHH, Lee IT, Liang K-W, Guo X, Rotter JI, Chen Y-DI, Kraus WE, Shah SH, Damrauer S, Small A, Rader DJ, Wulff AB, Nordestgaard BG, Tybjærg-Hansen A, van den Hoek AM, Princen HMG, Ledbetter DH, Carey DJ, Overton JD, Reid JG, Sasiela WJ, Banerjee P, Shuldiner AR, Borecki IB, Teslovich TM, Yancopoulos GD, Mellis SJ, Gromada J and Baras A. Genetic and Pharmacologic Inactivation of ANGPTL3 and Cardiovascular Disease. *N Engl J Med*. 2017;377:211-221.

66. Fernandez-Prado R, Perez-Gomez MV and Ortiz A. Pelacarsen for lowering lipoprotein(a): implications for patients with chronic kidney disease. *Clin Kidney J*. 2020;13:753-757.

67. Guardiola M, Exeter HJ, Perret C, Folkersen L, van’t Hooft F, Eriksson P, Franco-Cereceda A, Paulsson-Berne G, Palmen J and Li K. PLA2G10 gene variants, sPLA2 activity, and coronary heart disease risk. *Circ Cardiovasc Genet*. 2015;8:356-362.

68. Holmes MV, Simon T, Exeter HJ, Folkersen L, Asselbergs FW, Guardiola M, Cooper JA, Palmen J, Hubacek JA and Carruthers KF. Secretory phospholipase A2-IIA and cardiovascular disease: a Mendelian randomization study. *J Am Coll Cardiol*. 2013;62:1966-1976.

69. Holmes MV, Exeter HJ, Folkersen L, Nelson CP, Guardiola M, Cooper JA, Sofat R, Boekholdt SM, Khaw K-T and Li K-W. Novel genetic approach to investigate the role of plasma secretory phospholipase A2 (sPLA2)-V isoenzyme in coronary heart disease: modified Mendelian randomization analysis using PLA2G5 expression levels. *Circ Cardiovasc Genet*. 2014;7:144-150.

70. Davidson MH, Ballantyne CM, Jacobson TA, Bittner VA, Braun LT, Brown AS, Brown WV, Cromwell WC, Goldberg RB, McKenney JM, Remaley AT, Sniderman AD, Toth PP, Tsimikas S, Ziajka PE, Maki KC and Dicklin MR. Clinical utility of inflammatory markers and advanced lipoprotein testing: advice from an expert panel of lipid specialists. *J Clin Lipidol*. 2011;5:338-67.

71. Passacquale G, Di Giosia P and Ferro A. The role of inflammatory biomarkers in developing targeted cardiovascular therapies: lessons from the cardiovascular inflammation reduction trials. *Cardiovasc Res*. 2016;109:9-23.

72. Consortium TI-RMRAIRM. The interleukin-6 receptor as a target for prevention of coronary heart disease: a mendelian randomisation analysis. *Lancet*. 2012;379:1214-1224.

73. Danesh J, Kaptoge S, Mann AG, Sarwar N, Wood A, Angleman SB, Wensley F, Higgins JP, Lennon L, Eiriksdottir G, Rumley A, Whincup PH, Lowe GD and Gudnason V. Long-term interleukin-6 levels and subsequent risk of coronary heart disease: two new prospective studies and a systematic review. *PLoS Med*. 2008;5:e78.

74. Rosa M, Chignon A, Li Z, Boulanger M-C, Arsenault BJ, Bossé Y, Thériault S and Mathieu P. A Mendelian randomization study of IL6 signaling in cardiovascular diseases, immune-related disorders and longevity. *NPJ Genom Med*. 2019;4:23.

75. Zacho J, Tybjærg-Hansen A, Jensen JS, Grande P, Sillesen H and Nordestgaard BG. Genetically Elevated C-Reactive Protein and Ischemic Vascular Disease. *N Engl J Med*. 2008;359:1897-1908.

76. Ridker PM, Everett BM, Pradhan A, MacFadyen JG, Solomon DH, Zaharris E, Mam V, Hasan A, Rosenberg Y, Iturriaga E, Gupta M, Tsigoulis M, Verma S, Clearfield M, Libby P, Goldhaber SZ, Seagle R, Ofori C, Saklayen M, Butman S, Singh N, Le May M, Bertrand O, Johnston J, Paynter NP and Glynn RJ. Low-Dose Methotrexate for the Prevention of Atherosclerotic Events. *N Engl J Med*. 2018;380:752-762.

77. Reiner Ž, Sirtori CR, Banach M, Ruscica M and Sahebkar A. Methotrexate for Cardiovascular Risk Reduction: The Right Choice? *Angiology*. 2020;71:105-107.

78. Ridker PM. Canakinumab for Residual Inflammatory Risk. *Eur Heart J*. 2017;38:3545-3548.

79. Kraler S, Wenzl FA and Lüscher TF. Repurposing Colchicine to Combat Residual Cardiovascular Risk: The LoDoCo2 Trial. *Eur J Clin Invest*. 2020;50:e13424.

80. Ajala ON and Everett BM. Targeting Inflammation to Reduce Residual Cardiovascular Risk. *Curr Atheroscler Rep*. 2020;22:66.

81. Kocyigit D, Gurses KM and Tokgozoglu L. Anti-inflammatory therapy in atherosclerosis. *Front Biosci (Landmark Ed)*. 2020;25:242-269.

82. Ridker PM, Devalaraja M, Baeres FMM, Engelmann MDM, Hovingh GK, Ivkovic M, Lo L, Kling D, Pergola P, Raj D, Libby P and Davidson M. IL-6 inhibition with ziltivekimab in patients at high atherosclerotic risk (RESCUE): a double-blind, randomised, placebo-controlled, phase 2 trial. *Lancet*. 2021;397:2060-2069.

83. Patrono C. Fighting residual cardiovascular risk in stable patients with atherosclerotic vascular disease: COMPASS in context. *Cardiovasc Res*. 2017;113:e61-e63.

84. Gallone G, Baldetti L, Pagnesi M, Latib A, Colombo A, Libby P and Giannini F. Medical Therapy for Long-Term Prevention of Atherothrombosis Following an Acute Coronary Syndrome. *J Am Coll Cardiol*. 2018;72:2886-2903.

85. Hernández JL, Lozano FS, Riambau V, Almendro-Delia M, Cosín-Sales J, Bellmunt-Montoya S, Garcia-Alegria J, Garcia-Moll X, Gomez-Doblas JJ, Gonzalez-Juanatey JR and Suarez Fernández C. Reducing residual thrombotic risk in patients with peripheral artery disease: impact of the COMPASS trial. *Drugs Context*. 2020;9.

86. Mathewkutty S and McGuire DK. Platelet perturbations in diabetes: implications for cardiovascular disease risk and treatment. *Expert Rev Cardiovasc Ther*. 2009;7:541-9.

87. de Ferranti SD, Steinberger J, Ameduri R, Baker A, Gooding H, Kelly AS, Mietus-Snyder M, Mitsnefes MM, Peterson AL, St-Pierre J, Urbina EM, Zachariah JP and Zaidi AN. Cardiovascular Risk Reduction in High-Risk Pediatric Patients: A Scientific Statement From the American Heart Association. *Circulation*. 2019;139:e603-e634.

88. Amar J. Microbiota-Host Crosstalk: A Bridge Between Cardiovascular Risk Factors, Diet, and Cardiovascular Disease. *Am J Hypertens*. 2018;31:941-944.

89. Jones-O'Connor M and Natarajan P. Optimal Non-invasive Strategies to Reduce Recurrent Atherosclerotic Cardiovascular Disease Risk. *Curr Treat Options Cardiovasc Med*. 2019;21:38.
